# The demography of swiping right. An overview of couples who met through dating apps in Switzerland

**DOI:** 10.1371/journal.pone.0243733

**Published:** 2020-12-30

**Authors:** Gina Potarca

**Affiliations:** NCCR LIVES, Institute of Demography and Socioeconomics, University of Geneva, Geneva, Switzerland; University of Salamanca, SPAIN

## Abstract

Within the span of almost ten years, phone dating apps have transformed the dating scene by normalizing and, according to some voices, gamifying the digital quest for a partner. Despite amplified discussion on how swipe-based apps damage the fabric of intimate ties, scientific accounts on whether they have led to different relationship patterns are missing. Using 2018 survey data from Switzerland, this study provides a rich overview of couples who met through dating apps by addressing three main themes: 1) family formation intentions, 2) relationship satisfaction and individual well-being, and 3) assortative mating. The data indicate that in Switzerland, dating apps have recently taken over as main online dating context. Results further show that couples formed through mobile dating have stronger cohabiting intentions than those formed in non-digital settings. Women who found their partner through a dating app also have stronger fertility desires and intentions than those who found their partner offline. Generally, there are no differences between couples initiated through dating apps and those initiated elsewhere regarding relationship and life satisfaction. Though more data are needed to capture the full range of users’ romantic and sexual experiences, current results mitigate some of the concerns regarding the short-term orientation or the poor quality of relationships formed through mobile dating. Findings finally suggest that dating apps play an important role in altering couple composition by allowing for more educationally diverse and geographically distant couples.

## Introduction

Swiping right as the act of swiftly expressing preferences is now deeply ingrained in everyday language and cultural practice [1, 2]. The concept of swiping originates in the gesture that users of Tinder, Grindr or other phone dating applications based on geolocation matching need to engage in to register their (dis)like of a potential partner. Two individuals are matched and allowed to initiate communication on the app when they both swipe right on each other. Dating websites or platforms (e.g., Match.com, OKCupid), have been popular since the mid-90s. They allowed their subscribers, after having filled in lengthy information about themselves and their preferences, to browse and get in contact with prospective mates, based on search criteria and (though not always) personality matching. In the 2010s though, people’s use of the Internet as context for partner search surged, largely driven by the increased adoption of smartphone applications for dating [3]. Though dating platforms now also provide an app version of their website, swipe-based apps have a distinctive set of features, including lower costs (e.g., most apps and their basic features can be used for free, whereas communication on dating websites usually requires a paid membership), and a more simplified interface (e.g., personal profiles largely comprised of pictures). By eliminating lengthy questionnaires, self-descriptions, and personality tests that users of dating websites typically need to fill in to create a profile, dating apps are much easier to use [4]. Increased accessibility likely normalized the act of dating online, and opened up use among new demographic groups, particularly young adults [5, 6]. Using an app on a smartphone that is almost permanently active and within reach also led to a significant boost in the proximity and mobility of dating options [1, 7]. Since users can easily connect with partners in their immediate area, but also in other spaces as they move around, the tempo of interactions is greatly accelerated and choices considerably increased [7].

As the practice of using dating apps became progressively widespread, anecdotal knowledge on how they changed courtship and the nature of intimate ties also thrived. The proliferation of mobile dating, with its multitude of partnering possibilities and a selection process largely based on visual content, is allegedly causing a “dating apocalypse” [8], turning individuals into “sexual freelancers” [9], lowering the quality of connections, and even threatening the mere existence of long-term commitment. Despite amplified media attention, we are yet to have nationally representative evidence on whether relationships initiated on swipe-based apps are different compared to relationships started in other contexts. Till now, surveys that measured where couples met have been scarce, and when such data existed, the sample of couples formed through dating apps was usually small [10]. Filling in this gap, this study reports findings based on a 2018 nationally representative Swiss survey and a sample of 3,245 respondents who met their partner in the last ten years. Given the launch of Grindr (one of the most emblematic dating apps for sexual minorities) in 2009, and of Tinder in 2012, this time frame neatly covers the post-dating app period. The paper centers around three main questions: 1) Are individuals in relationships formed through swipe-based apps less interested in family formation: do they have lower intentions to form long-terms unions (e.g., marriage, cohabitation), and are they less interested or intent on becoming parents? 2) Are unions initiated through phone apps less satisfying, and are they associated with lower levels of subjective well-being? And finally, 3) are they more exogamous in terms of education, origin (e.g., migration background), geographical location, or age? By addressing several partnership themes (e.g., commitment, quality, sorting), this study provides a rich descriptive account of couples who met through swipe-based apps. To understand whether this dating medium brought about greater transformations in the demography of couples than previous digital modes of mate selection [10, 11], couples that met via phone apps are compared to those formed offline (i.e., in non-digital settings), as well as those initiated through dating platforms and other digital spaces such as online social networks. Furthermore, to explore selection into mobile dating and scrutinize whether the patterns found for prevailing couples correspond to singles’ values and intentions, the analysis is supplemented with an investigation of partner-seeking users of dating apps based on 2018 data from the Swiss Household Panel. Focusing on Switzerland, with its enduring conservatism in family ideology and the dominance of marriage as family model [12], has the advantage of making it easier to notice potential deviations in the outlook of partnerships that mobile dating may have encouraged.

### Do dating apps facilitate relationships less oriented towards long-term commitment?

Recent decades have witnessed increasingly rapid and complex transformations in marriage and family patterns, including a rise in childlessness and non-marital cohabitation [13]. Family theorists have tried to capture the essence of such changes under broad theoretical constructs. Contemporary romance in Western societies is allegedly undergoing “a brave new world of intimacy” [14], or a “deinstitutionalization” of marriage [15]. Scholars also speak of a new culture of courtship and “hooking up” [16], referring to sexual practices that lack legal bindings, or a clear-cut set of rules and expectations. In removing the obstacle of physical distance and allowing individuals to disengage from bonds with astonishing ease and minimal costs, online partner search tools such as dating platforms, are presumably contributing to the increase in fleeting connections devoid of commitment [17]. Through the many unique advantages that they afford (e.g., immediacy, proximity, surplus of choice), some consider dating apps to mark a significant leap forward in virtual dating technologies, with an even greater power to accelerate these trends [18]. First, it is argued that the casual dating mindset promoted on apps encourages a superficial and consumerist approach to finding a match, inciting an objectification of partners and a focus on visual information only [2, 7]. Second, the card-game resemblance of the interface and its swipe-based logic create the setup of a game played at high speed, with a constant pursuit of the next best thing [2, 4]. We would thus notice less interest in family formation not only among users of dating apps, but also among established couples, given that the search context people opt into sets the course for whether they pursue short- or long-term mating [19, 20], and evidence showing that the objectification of one’s romantic partner, i.e., the presumed bedrock of mate selection on dating apps, is negatively associated with relationship commitment [21]. Individuals in unions resulting from dating apps would therefore have lower intentions to marry or move in together (if in non-residential partnerships), and less plans or desires to have (a) child(ren) in the near future compared to those in unions formed offline or in other online meeting places. Less interest in family formation should be particularly visible when comparing dating apps to dating platforms. The latter are typically marketed towards long-term matching; its users state clear family plans and intentions, and often scan the profiles of candidates on fertility intentions. As opposed to dating apps, traditional desktop-based dating platforms are also more restrictive and allow for less spontaneity of use [22], creating fewer incentives for short-term dating. Confirming the discrepancy between the two modes of digital dating, research looking at the transition from web to mobile dating (i.e., among users adopting the mobile application of the dating service) showed an increase in contact between users, as well as a surge in impulsivity (i.e., responding to messages without checking the profile of those who contacted them) [23]. Faced with a multitude of choices, dating app users are not only more likely to reject potential partners [24], but also less likely to make well-thought out decisions [25].

Nevertheless, the tendency that users have to frame dating apps as tools geared towards casual rather than serious dating could also be part of a discourse meant to attenuate the lingering stigma of seeking love online [2, 26], and may not reflect true partnering intentions. Those in pursuit of long-term engagements, particularly women [27], might even take advantage of the surplus of alternatives easily available on dating apps (see the over-representation of single men using phone apps in S5.1 Table in S1 File) to find long-term-oriented partners. In this case, the data might reveal that individuals who met their partner through a dating app have similar or even higher family formation intentions compared to those who met theirs elsewhere.

### Are relationships formed via dating apps less satisfying?

Could an image-based selection impact the quality of relationships formed through dating apps or partners’ broader subjective well-being? Phone apps not being known as intermediaries for serious dating [2, 26], as well as the particularities of their photo-centric interface, might mean that users do not pay a lot of attention to aspects conducive to a good match, such as compatibility in interests, values, or personality [28]. Aware of the hook-up ethos of dating apps and overwhelmed by the abundance of options, some users have actually expressed reluctance regarding the authenticity and quality of connections established while swiping [29]. Assuming that visual assessment plays a major role in how app users select their partner, and given that partners objectifying each other usually experience lower relationship satisfaction [21, 30], couples initiated on phone dating apps are likely less satisfied with their union than those formed in other settings. In contrast to dating apps, online dating platforms are designed and advertised for the precise purpose of facilitating compatibility-based matches [31]. The more complex interface of dating websites allow for richly detailed information about prospective mates, as well as options to filter and select candidates along key socio-demographic attributes or preferences [32]. Through these unique features, dating websites are likely to assist people in finding more suitable partners than dating apps. Research found that, in the U.S., couples formed through dating websites were linked to greater marital satisfaction than those formed offline or through other online venues [33]. One could therefore expect that relationships built through dating apps are associated with lower relationship satisfaction and lower subjective well-being than unions formed in either face-to-face or virtual settings, particularly those formed on dating platforms. Nevertheless, since there is also evidence showing that relationship satisfaction is only marginally connected to how couples met [11], it could also be expected that dating apps have little effect on partnership quality.

### Is meeting through dating apps linked to more exogamy?

The final question is whether dating apps affect the way people sort into partnerships. Does a large and more easily accessible supply of potential partners, as well as an appeal to novel audiences [6], make dating apps a virtual social space more likely to encourage exogamy? Empirical studies have found that Internet-matched heterosexual unions display less within-couple similarity in terms of education, race or religious background [10, 34], especially compared to unions created in typically homogenous settings, such as school, circles of friends, or family [35]. Most studies, however, were unable to single out the specific effect of dating websites or apps on exogamy, and largely focused on couples formed via the Internet in general [34], or through dating websites and apps jointly considered [10]. It could be expected that through more democratized use, dating apps provide exposure to an even greater socio-demographic diversity than dating platforms or other online settings such as social networks. The latter usually accommodate pre-existing social ties and are likely to reproduce a level of segregation and ultimately endogamy similar to offline networks [10]. Based on the geolocation matching of people in spatial proximity, dating apps may also facilitate contacts between people located in often-segregated spaces (ib.). Nevertheless, the option of setting the location radius to wider areas, as well as the mobility afforded by smartphones, opens up the possibility of matching with potential candidates embedded in other circles. One direct consequence would be an increase in geographical exogamy. Facilitating encounters between geographically distant partners, dating apps likely produce more long-distance non-residential relationships than other offline and online meeting contexts. A second indirect consequence of enlarging the dating pool could be an increase in socio-demographic exogamy. Access to a wider and more socially diverse partnership market generates more chances for partnering across different groups [36]. In addition to structural arguments, the use and availability of information on potential partners may also play a role. As already stated, the initiation of contact on dating apps relies more on aspects linked to physical appearance [7], and less on textual descriptions or information on income, racial background, profession, etc., often mandatory on dating platforms as part of standardized profile creation. Even though apps such as Tinder recently changed their interface to allow users to include education and work information on their profiles, it is often optional, and does not change the reliance on pictures as the main criterion informing partner selection [7, 37]. The emphasis on visual display likely encourages app users to make decisions based on a more instinctive rather than a thoroughly informed evaluation of candidates [2]. We should thus observe greater socio-demographic exogamy (on aspects such as education, migration background, or age) among couples formed through dating apps than among couples formed in other face-to-face or online contexts. Although previous research has shown that partners who met via the Internet (through dating websites and apps, combined) are closer in age than those who met offline [10], it can be expected that phone dating apps encourage greater age exogamy than dating websites simply due to a wider age range of users. Phone apps are popular among adults in their 20s as well as those over 30, whereas websites largely attract people over 40, as seen in the sample of partner-seeking singles (see S5.1 Table in S1 File). A significant age gap between partners however could take the form of either female hypogamy (i.e., the woman is the older partner) or female hypergamy (i.e., the man is the older partner). Whereas the former could be viewed as disruptive of gendered norms of partnering [38], the latter is suggestive of social closure and gender inequality [39], insofar as age hypergamy is still linked to status hypergamy [40]. Given the assumption that people, especially women, have greater freedom to create less socially constrained identities online [41], it was initially predicted that Internet dating might challenge gendered courtship behaviors. Existing research nevertheless shows that online interactions still follow traditional scripts of partner selection [42], including age‐hypergamous choices.

## Selection into mobile dating

In an experimental design framework in which single adults are randomly assigned to a treatment group (i.e., use of dating apps) and a control group (i.e., non-use of dating apps), one would be able to draw strong conclusions about the causal effects of using phone dating apps on relationship patterns. With the observational data at hand, however, we can make inferences of association at the level of the population, but there is a high risk of endogeneity and selection bias affecting results. One important concern is that users of phone dating apps may be systematically different from non-users in terms of both observable and unobservable characteristics, which may influence the type of relationships they establish.

There are several potential sources of selection bias. First, people choosing a certain type of virtual dating tool may have different partnering intentions, values, or readiness to commit; this may have little to do with the dating environment itself. For instance, people holding less traditional family values may be both more likely to be selected into dating app use (with its modern features), and less likely to pursue conventional family forms (i.e., marriage, parenthood). Though the main preconception is that Tinder and similar apps attract users searching for sexual partners only [7], research into the motivations for using dating apps revealed a wide variety of reasons, ranging from desire for casual sex to (and thus not excluding) the pursuit of long-term relationships [26, 43, 44]. There also seems to be no difference between app users and dating website users, and even when present (e.g., app users are more sexually permissive), the difference is fully accounted for by gender [45] or age [5]. Age or life course stage may also be a confounding factor as it is associated with the exposure to treatment (i.e., younger people are more likely to use phone dating apps), as well as readiness to invest in a long-term union [46, 47].

Second, singles who choose mobile dating may have particular psychological features that affect the way they form and construct relationships. Certain personality dimensions, such as extraversion or internal locus of control (i.e., the belief that one is in charge of life events and outcomes, as opposed to outside forces), are known to impact mate selection, short-term mating, and marital quality [48–54]. Individuals that resort to a more agentic way of selecting partners, one that also involves constant interactions with others, may be particularly open, extroverted, and may have a high internal locus of control. Since research does seem to suggest that app users are more extroverted and open to new experiences than non-users [55], a comparative study of relationships initiated through phone apps and other settings needs to acknowledge differences in psychological profile.

Finally, there may be structural reasons that influence both the use of phone dating apps and relationship outcomes, particularly exogamy. For instance, limited time to search for partners offline (given job constraints) or a diminished dating pool (due to a narrow social circle, or living in less populated areas) may push singles not only to try out different strategies of partner search, but also to broaden their mating preferences [56]. When faced with a deficit of potential partners, people’s willingness to extend their search radius beyond their (often segregated) social space increases, leading to greater social and cultural mixing [36]. Time pressure and local marriage market conditions may affect people’s partner search process, as well as their progression into more committed relationship forms [57, 58].

In the absence of repeated-measure data allowing to control for time-constant heterogeneity without observing it, this study attempts to minimize selection bias by employing several strategies. First, all the analyses control for a comprehensive set of variables that may moderate the relationship between app use and relationship patterns, such as age group, previous marital or parenthood experience, time pressure, and type of residential area (see the following section). Second, I conduct a propensity score analysis [59] to examine if comparison groups (e.g., respondents who met their partner via dating apps versus respondents who met their partner offline) are matched on key observed covariates. Third and finally, I focus on the pre-partnering stage and examine several attitudinal, psychological, and structural characteristics of singles using dating apps in a complementary analysis. This strategy allows for a direct comparison between individuals exposed, albeit non-randomly, to the treatment condition and those in the control group.

## Materials and methods

### Data

Data for this analysis are drawn from the 2018 Family and Generations Survey (originally Enquête sur les familles et les générations (EFG) 2018), conducted by the Swiss Federal Statistical Office (FSO). The survey is part of the federal population census program and aims to provide data on the current state and evolution of families, and more generally on the relationship between generations. It targets the permanent resident population living in private households, aged 15 to 79 years old, and is scheduled to take place every five years, starting with 2013. The 2018 survey is the first to include a measure on where couples met. The data were collected through computer assisted telephone interviews (CATI), followed in most cases by additional online or paper questionnaires (CAWI/ PAPI). The latter included the item on where couples met. Respondents who filled in the CATI questionnaire were invited to participate in the CAWI/ PAPI follow-up survey via e-mail, and in the case of refusal, through paper questionnaire sent via post. Those who responded at the CAWI/ PAPI survey are thus a sub-sample (approximately 90%) of those who responded at the CATI questionnaire. The interviews were held in three languages: German (standard German or Swiss German), French, and Italian. The survey included an oversampling (i.e., doubling) of persons older than 24 with (a) child(ren) under 13 in the household. To account for this, I apply survey weights, as well as control for age and presence of children in multivariate models.

From an initial random sample of 36,029 persons, 16,815 (47%) participated in the survey. From this sample, I excluded respondents who did not participate in the follow-up CAWI/ PAPI survey (n = 1,587), those without a partner (*n* = 2,929), as well as partnered participants without an answer on where they found their match (*n* = 212). The survey identified the presence of a residential partner by first asking about specific household members and their relationship to the respondent. Second, if respondents did not indicate a partner they live together with, they were asked the following: “Do you have an intimate relationship with a person who does not, or only partially, live in your household?” I furthermore removed cases with missing information on marital status (*n* = 2), and respondents younger than 18 (*n* = 54). Given convergence in partners’ attributes over time (e.g., in addition to assortative selection, partners can influence for instance each other’s educational achievements [60]), and the fact that partnerships initiated offline are disproportionately longer in duration, the analyses of exogamy risks over-estimating dating app effects. To balance the sample of partnerships in terms of duration, and to also capture couples that met in the post-app dating era, I excluded relationships lasting longer than ten years (*n* = 8,786). To further counter concerns regarding the differential survival of relationships formed through dating apps, supplementary analyses (see S6 Section in S1 File) looked at relationships established in the last five years, with results largely similar to those reported here. The final sample used in the analyses included *3*,*245* partnered individuals, of whom 104 (4.12%—weighted by wecritpers) used dating apps, 264 (7.33%) used dating websites, and 125 (5.24%) used other online services to find their partner in the last ten years. S1 Table in S1 File provides a socio-demographic overview of the sample.

### Measurements

To measure *marriage intentions*, respondents who had a non-marital partner were asked, “Do you intend to marry your partner within the next two years?”, with the following answer possibilities: “yes”, “probably yes”, “probably not”, “no”, and “do not know”. I considered respondents who answered “yes” or “probably yes” as having strong marital intentions. Those who responded “probably not,” “no,” or “do not know” were classified as having no (or weak) marital intentions. I applied the same coding for *cohabiting intentions*, which is based on the item “Do you intend to move in with your partner within the next two years?”. The question was addressed to respondents over 20, who had partners living entirely or partially outside their household. *Fertility desire* was captured via an item asking “Would you want to have a(nother) child?”, with the answer options “yes” or “no”. If the female respondent was pregnant, the question referred to having another child in addition to the one expected. *Fertility intentions* were based on the question “Do you wish to have your first/ next child within the next three years?” The survey allowed for the following possible answers: “yes, certainly,” “yes, probably,” “no, probably not” “no, certainly not,” and “do not know”. As with marital and cohabiting intentions, I grouped respondents who chose either one of the first two categories as having strong fertility intentions, and those who chose the last three as having no (or weak) fertility intentions. The two questions related to fertility were addressed to women under 50, men who have a female partner under 50, and men under 60 with a same-sex partner.

To measure *relationship satisfaction*, respondents provided answers on a scale from 0 (“not at all satisfied”) to 10 (“very satisfied”) to the following question: “To what extent are you satisfied with the relationship with your partner?” *Life satisfaction* was measured on a similar 0–10 scale through the item “In general, how satisfied are you with your life currently?”.

Furthermore, *educational exogamy* was operationalized as partners having different levels of education. Educational level was measured via the question “What is the highest level of education you (your partner) have completed by obtaining a certificate or diploma?”. Possible answers included: 1) “primary compulsory school”, 2) “vocational secondary school”, 3) “academic secondary school”, 4) “upper secondary education and non-tertiary post-secondary education”, 5) “tertiary education (i.e., applied university or academic university degree, and further post-graduate degrees)”. The item referring to partner’s education was only asked to respondents with partners over 25. Given the under-representation of respondents with primary education among those who met their partner through dating apps (*n* = 5), the final coding for the educational level of respondents and their partners dichotomously distinguishes between those with tertiary education and those with non-tertiary education. Educational exogamy thus denotes couples that include one partner who did not graduate from university and another who did.

I further constructed a measure of *exogamy on migratory background* (or origin) by looking at both partners’ migration profile. To identify whether someone was a Swiss native or a migrant, I used information on current nationality, nationality at birth, and country at birth. If someone had current Swiss nationality, and was born Swiss, irrespective of country of birth, they were coded as ‘native.’ If they currently had a foreign nationality or were currently a Swiss national, but had a foreign nationality at birth, they were coded as migrants. If the respondent and their partner had a different migration profile, the couple was considered exogamous. For partner’s nationality at birth, the survey only distinguished between Swiss and (broad) foreign nationality. In the absence of foreign partner’s specific nationality at birth, the study only looks at exogamous unions between Swiss natives and migrants, ignoring exogamous matches between migrants belonging to different nationality groups. Nevertheless, given that inter-partnering between immigrants and the native born remains a key measure of immigrant social integration [61], its significance is greater than inter-partnering between immigrants from different groups.

*Age difference* applies only to heterosexual couples and similar to other research [62], distinguishes between “age homogamy” (the male partner is 0–2 years older than the female partner), “age hypergamy” (the man is at least 3 years older), and “age hypogamy” (the woman is at least 3 years older). Finally, *geographical exogamy* was created using information on how much time (in minutes) it takes the respondent to reach their partner’s residence. The question was addressed to those older than 20, with a partner who did not live in the same household. Respondents were encouraged to provide an estimate of their door-to-door travel time via a commonly used transportation mode. Based on their answers, I created a three-category variable of geographical distance, distinguishing between: “short distance” (under 30 minutes of traveling time), “moderate distance” (between 30 minutes and one hour), and “long distance” (more than one hour).

**Fig 1:**
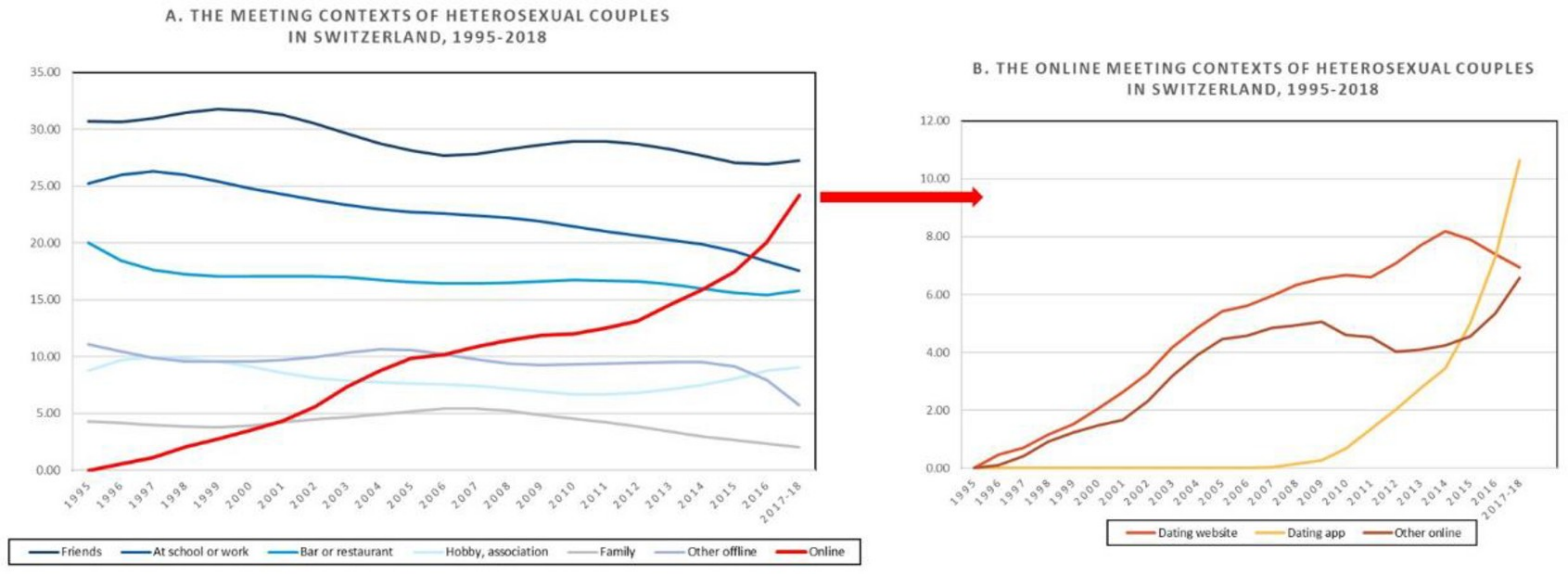
How heterosexual couples met in Switzerland, by year of meeting (1995–2018): all contexts (Panel A), specific online contexts (Panel B). Source: EFG 2018. Weighted data by wecritpers. Lowess regression smoothing with bandwidth = .6 is applied, except for online meeting contexts, where a bandwidth = .3 is applied. Percentages before applying lowess smoothing are reported in S2 Table in S1 File.

The main independent variable concerns the context in which partners met. The survey asked “How did you meet your partner?”, and allowed for a single answer among several options, including: 1) “through friends or acquaintances”; 2) “through family”; 3) “at school, through studies, at work”; 4) “in a bar, a disco, a concert, a neighborhood party, …”; 5) “through a hobby, association, sports’ club”; 6) “on a dating website (for example eDarling, Parship, Swissfriends)”; 7) “through a dating app on the smartphone (for example Tinder, Lovoo, Grindr)”; 8) “through a social network, chat room, or other online services (for example Facebook, Instagram, Twitter)”; or 9) “other”. Except when integrated into a descriptive graph illustrating the evolution of how couples met in Switzerland over time (Fig 1), all non-digital settings were grouped into one “offline” category. The final operationalization of couples’ *meeting context* included the following four categories: 1) “offline”; 2) “dating app”; 3) “dating website”; 4) “other online”. Though not verifiable, given the examples used in its description, the last category most likely refers to meeting through online social networks.

The analyses also accounted for several control variables, such as: being part of a same-sex couple (excluded in the analysis of marital intentions as same-sex couples do not yet have a legal option to marry in Switzerland); age, measured in categories (18–29, 30–39, and over 40) to avoid collinearity with partnership duration (measured in years); and type of union (with options: “marriage”, “cohabitation”, or “non-residential partnership”). Same-sex couples, but not opposite-sex couples, in Switzerland currently have the possibility of entering registered partnerships; therefore, for heterosexual couples, cohabitation refers to co-residence without any legal implications. Given the importance of socio-economic resources for partnering transitions and expectations [63, 64], in addition to education, I added a measure of employment. This distinguished between being employed (grouping respondents who are salaried employees, independent, working for the family business, or in apprenticeship), or out of employment (grouping respondents who are in unemployment, training, retirement, those who have a disability, homemakers, or others). To account for the potential impact of prior relationship and fertility experience on family formation intentions or assortative mating [65, 66], the study controlled for whether the respondent was previously married; previously cohabited with another partner (irrespective of their cohabitation leading to marriage); has any (living) biological children, or any children in common (including an ongoing pregnancy) with the current partner. Macro- or micro-level structural factors, particularly relevant in the analysis of exogamy, such as work-life conflict or type of region (distinguishing between “densely populated” areas, “moderately urbanized”, “or sparsely populated”) were also included. As sparsely populated regions in Switzerland have a low level of socio-demographic heterogeneity (with an over-representation of Swiss native residents and adults with non-tertiary education), the densification measure also captures the diversity of daters’ local marriage market. To create a measure of work-life conflict, I constructed an aggregated score (Cronbach’s alpha 0.796) based on four items describing the frequency of experiencing each of the following in the last twelve months: “returning from work too tired to do some of the household chores”, “having difficulty fulfilling family responsibilities because of work”, “having difficulty concentrating at work because of family responsibilities”, and “having difficulty coordinating different activities”. There were six response categories: 1) “never”, 2) “rarely”, 3) “sometimes”, 4) “most of the time”, 5) “always”, and 6) “not concerned”; the first and final categories were grouped together.

Considering the alignment between family behavior and family values [67, 68], the expectation that dating apps facilitate less committed relationships may be particularly valid among less traditionally oriented individuals. Given the risk of endogeneity (i.e., the possibility that partners adjust their values to match behavior post-union), I do not include a direct measure of attitudes towards marriage and family. Instead, I focus on the degree of religiosity, which is a more stable aspect of social and individual identity [69, 70] and is strongly associated with traditional marriage orientation [71]. Religiosity was measured via the item “Regardless of whether or not you belong to a church or a religious community, would you say that you are: 1) not at all religious, 2) rather not religious, 3) rather religious, and 4) very religious.” The first two categories are combined into a “non-religious” group, and the latter two into a “religious” group.

For the analyses modeling relationship and life satisfaction, given the link between perceived health and subjective well-being [72], I added a measure of self-rated health. Based on an item asking “How is your state of health, in general? Is it: 1) very good; 2) good; 3) rather good; 4) bad; 5) very bad”, I grouped the last three categories to indicate poor health. In the analysis of family formation intentions, due to the importance of partnership quality for relationship progression [64], I added a measure of relationship satisfaction. Finally, other controls that were included but proved insignificant are linguistic region, geographical distance between partners (when modeling cohabiting intentions), and whether respondents experienced parental separation.

## Results

### How couples met in Switzerland

First, the study reports regression-smoothed percentages of where heterosexual couples met in Switzerland over time (Fig 1, Panel A). The number of same-sex couples in the sample is too small (*n* = 23) to allow for a similar investigation. To follow the evolution of online meeting contexts starting with the initial rise in dating platforms in the mid-90s, this graph also includes couples that met between 1995 and 2007 in addition to those included in the main analysis (i.e., who met between 2008 and 2018). For the purpose of drawing comparisons with trends occurring among heterosexual couples in the U.S. [3], offline contexts are separately considered, and digital meeting places are grouped in one “online” category. Due to a small number, couples that formed in the year of survey (i.e., 2018) are combined with couples formed in the previous year. We notice that meeting through friends remains the main way that couples meet in Switzerland. Nevertheless, this social intermediary is in slow decline, as are other offline meeting places, particularly meeting through school, work, or family. What is rapidly increasing is meeting on the Internet, with a quarter of relationships initiated in the last two years having started online. Similar to Rosenfeld et al. [3], the curve for online dating describes an initial steady surge in popularity in the mid-90s, followed by a plateau starting mid-2000s, and then a more recent accelerated growth after 2012. These curves also indicate the transition from networked ways of selecting a partner to mate selection that essentially involves connecting with strangers, a trend alluding to the privatization of partner selection in both the U.S. and Switzerland. As opposed to the U.S., however, meeting online has not yet overtaken meeting through friends, which is suggestive of Switzerland’s greater population density as well as people’s stronger attachments to local informal ties [73]. Panel B of Fig 1 breaks down the ‘meeting online’ curve into specific contexts of online selection. We see that the initial rise in the Internet as context of matching is attributed to the use of dating websites, alongside other digital contexts such as online social networks, whereas the recent surge has been driven by the popularity of dating apps. In fact, after 2016, the latter took over as the main online dating context in Switzerland.

### Propensity score estimation

Given differences in socio-demographic profile across meeting context (see the *Descriptive Statistics* section in S1 File), and the cross-sectional nature of the data, it is essential to check whether observed covariates are reasonably equally distributed between treatment (i.e., respondents who met their partner via dating apps) and comparison (i.e., respondents who met their partner offline) groups. To address potential confounding due to observed selection bias, I use Stata’s *pscore* [74] command to calculate a propensity score [59] based on a logit regression estimating the probability of treatment and including all previously noted covariates. The algorithm split the sample by quintiles, and, within each quintile, it tested whether the average propensity score differed in the treatment and comparison groups. The splitting continued until the program identified the smallest number of blocks where, for each block, the propensity score mean was equal across the two groups. In this case, the final number of blocks was five. The algorithm then continued by testing the balancing property for each covariate. The result of this test revealed that, for the current sample, the balancing property was satisfied, meaning that the propensity score had a similar distribution across respondents who used dating apps to find a partner and those who found their partner offline. The same outcome was reached when comparing the former to respondents who found their match through a dating website. Given that these tests indicate balance in terms of observed covariates, the multivariate analyses that follow are based on an unmodified sample. Nevertheless, it is acknowledged that balance in measured variables does not indicate balance in unmeasured variables, and that it is still possible for residual confounding due to unobserved characteristics to occur.

### Multivariate results

To examine if individuals who met their partner through dating apps are less focused on long-term commitment than those who met their partner elsewhere, a series of logistic regression models of having strong family formation intentions, accounting for an extensive set of covariates, are presented in Table 1. The results indicate that respondents who met through a dating app do not differ significantly in terms of marital intentions, fertility desire or fertility intentions from those who met their partner offline. Nevertheless, non-residential couples formed through dating apps have significantly stronger intentions to move in with their partner than those who met offline. Comparing dating apps to the other two online categories revealed no significant differences. In additional analyses (not shown), I estimated ordered logistic regression models of family formation intentions operationalized on the original 5-point scale (see Measurements sub-section). The results were similar regardless of analytic strategy.

**Table 1:** Logistic regression coefficients predicting family formation intentions by meeting context.

	Marital Intentions	Cohabiting Intentions	Fertility Desire	Fertility Intentions
	Coeff. (SE)	Coeff. (SE)	Coeff. (SE)	Coeff. (SE)
Meeting context (ref.: offline)				
Dating app	0.034	1.353**	0.392	0.481
	(0.308)	(0.472)	(0.309)	(0.322)
Dating website	−0.100	0.505	−0.143	0.259
	(0.361)	(0.352)	(0.231)	(0.231)
Other online	0.581	0.758	0.187	0.559†
	(0.521)	(0.508)	(0.337)	(0.326)
Pseudo R-squared	0.166	0.193	0.361	0.122
*N*	*1*,*908*	*928*	*2*,*651*	*2*,*649*

Source: EFG 2018.

Note: Weighted data by wecritpers. The models control for: gender, (same-sex couple), (type of union), age, tertiary education, whether employed, whether previously married, prior cohabitation, biological children, (common children), religiosity, migration background, work-life conflict, relationship satisfaction, and partnership duration. Covariates within parenthesis are not included in all the analyses (e.g., same-sex couple not included in the model of marital intentions). The full list of regression coefficients is provided in S3.1 Table in S1 File.

† *p* < .10

** *p* < 0.01.

**Fig 2:**
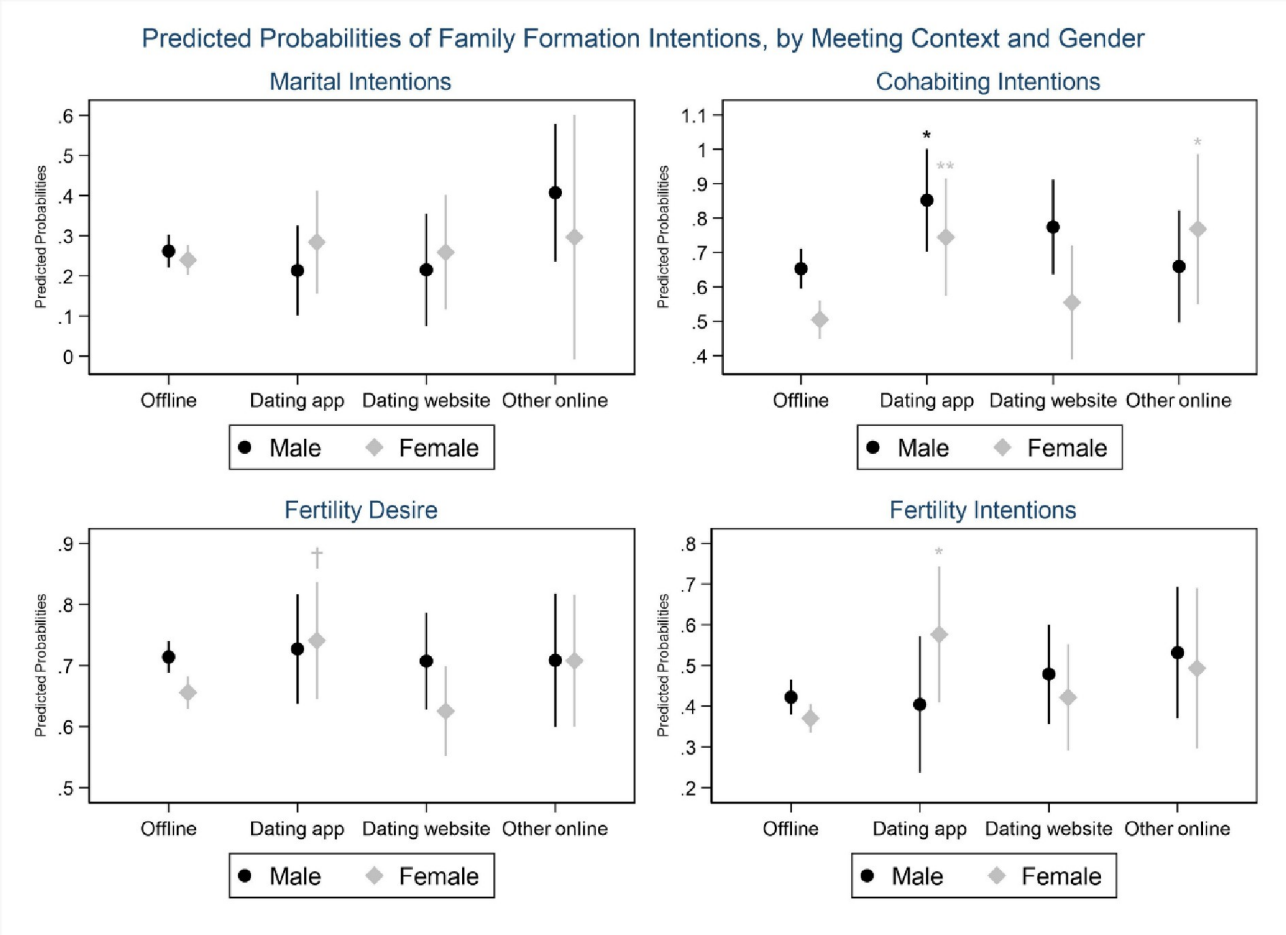
Predicted probabilities of family formation intentions, by meeting context and gender, 95% CI. Based on logistic regression models including interactions between meeting context and gender, controlling for whether same-sex couple, type of union, age, tertiary education, whether employed, whether previously married, prior cohabitation, biological children, (common children), religiosity, migration background, work-life conflict, relationship satisfaction, poor health, and partnership duration. Stars indicate probabilities significantly different compared to the offline category: † *p* < 0.10; * *p* < .05; ** *p* < 0.01.

To explore whether there is gender variation in the link between meeting via dating apps and long-term commitment, Fig 2 reports predicted probabilities of family formation intentions by meeting context and gender, with all other variables held at mean value. The graph corresponding to marital intentions indicates that neither men nor women who met their partner through a dating app have significantly lower intentions to marry in the next two years than those who met their partner offline. Nevertheless, both men and women who met their match on a dating app, as well as women who found their partner via other online venues, have stronger cohabiting intentions than those who met their partner in non-digital settings. Finally, we notice that women who found their partner through a phone app have a marginally stronger desire to have children (*p* = .096) and significantly more pronounced intentions to have children in the next three years (*p* = .018) than those who met their partner offline.

To assess if relationships initiated on dating apps are linked to lower partnership satisfaction and individual well-being compared to relationships started elsewhere, especially on dating websites, Table 2 reports OLS regression coefficients predicting relationship and life satisfaction. The results indicate no significant difference between unions started offline and those started through dating apps. Additional models comparing couples who met through dating platforms and those who met through dating apps also reveal no significant differences. Nevertheless, findings from an extra analysis across union type (see S4.1 Fig in S1 File) showed that individuals in non-residential couples initiated on dating platforms are significantly more satisfied than those in non-residential couples initiated offline. Furthermore, individuals who met their non-residential partner via phone apps are less satisfied than those who met theirs through dating websites (*p* = .018).

**Table 2:** OLS regression coefficients predicting relationship and life satisfaction by meeting context.

	Relationship Satisfaction	Life Satisfaction
	Coeff. (SE)	Coeff. (SE)
Meeting context (ref.: offline)		
Dating app	−0.171	−0.228
	(0.218)	(0.220)
Dating website	0.190†	0.080
	(0.115)	(0.125)
Other online	−0.303	−0.462*
	(0.253)	(0.220)
R-squared	0.088	0.203
*N*	*3*,*206*	*3*,*210*

Source: EFG 2018.

Note: Weighted data by wecritpers. The models control for: gender, same-sex couple, type of union, age, tertiary education, whether employed, whether previously married, prior cohabitation, biological children, religiosity, migration background, work-life conflict, poor health, and partnership duration. The full list of regression coefficients is provided in S3.2 Table in S1 File.

† *p* < .10

* *p* < 0.05

Finally, to test whether dating apps are associated with greater exogamy than other meeting contexts, Table 3 first presents the results of three logistic regression models predicting educational exogamy, exogamy on origin (i.e., migration background) among Swiss natives, and exogamy on origin among migrants, respectively. The table then also reports findings for two multinomial logistic regression models predicting first, the age difference between partners, and second, geographical exogamy. All models controlled for exposure to offline marriage markets by including indicators of population density and time availability (proxied by work-life conflict).

**Table 3:** Logistic and multinomial logistic regression coefficients predicting exogamy by meeting context.

	Educational Exogamy	Exogamy on Origin among Natives	Exogamy on Origin among Migrants	Age Difference (Ref.: Age Hypergamy)		Geographical Exogamy (Ref.: Short Distance)	
	Logit	Logit	Logit	Multinomial Logit		Multinomial Logit	
				Age Homogamy	Age Hypogamy	Moderate Distance	Long Distance
	Coeff. (SE)	Coeff. (SE)	Coeff. (SE)	Coeff. (SE)	Coeff. (SE)	Coeff. (SE)	Coeff. (SE)
Meeting context (ref.: offline)							
Dating app	0.650*	0.228	0.641	−0.327	−0.143	0.968*	1.062*
	(0.653)	(0.315)	(0.349)	(0.328)	(0.414)	(0.457)	(0.483)
Dating website	0.010	0.237	0.347	0.511**	−0.190	0.571	1.394**
	(0.354)	(0.209)	(0.257)	(0.192)	(0.337)	(0.430)	(0.439)
Other online	−0.260	1.314***	−0.202	0.061	−0.398	−0.120	0.690
	(0.394)	(0.295)	(0.355)	(0.302)	(0.520)	(0.584)	(0.478)
(Pseudo) R-squared	0.036	0.078	0.064	0.048		0.084	
*N*	*2*,*801*	*1*,*955*	*1*,*240*	*3*,*156*		*925*	

Source: EFG 2018.

Note: Weighted data by wecritpers. The models control for: gender, (same-sex couple), (type of union), age, tertiary education, whether employed, previously married, prior cohabitation, biological children, religiosity, (migration background), type of residential region, work-life conflict, and partnership duration. Covariates within parenthesis are not included in all the analyses (e.g., same-sex couple not included in the model of age difference). The full list of regression coefficients is provided in S3.3 Table in S1 File.

* *p* < 0.05

** *p* < 0.01

*** *p* < 0.001.

First, when it comes to educational assortative mating, results indicated that meeting through dating apps was indeed associated with greater exogamy (i.e., couples are more likely to include a partner with tertiary education and one with non-tertiary education) than meeting offline. The effect is largely driven by tertiary-educated women partnering down (see S4.2 Fig in S1 File). An additional model employing the detailed categorization of offline meeting contexts (results in S4 Table in S1 File) revealed that dating apps have an enhancing effect on educational exogamy when compared to most offline settings, especially meeting at school or work, through a hobby or association, or via friends.

Second, irrespective of migration background, meeting through dating apps had no effect on the likelihood of being part of an exogamous couple including a native and a migrant partner. Among natives, finding a partner online was related to greater exogamy on origin only when having used other digital tools such as online social networks.

Furthermore, results showed that couples who met through dating apps were not significantly different from those who met offline in terms of the age difference between partners. Couples initiated through dating platforms were, however, closer in age than those initiated offline. Additional analysis including an interaction between meeting context and age group (predicted probabilities graphed in S4.3 Fig in S1 File) indicated that dating websites encourage age homogamy for respondents over 30, and that respondents between 18 and 29 years old who met their partner through dating apps are less age-homogamous but more age-hypergamous than those who met offline.

Lastly, results for geographical exogamy revealed that respondents who met their match via dating apps had to travel significantly greater distances (both moderate and long) to see their partner than those who met offline. Dating platforms do not seem to promote significantly more moderate-distance relationships, but they do have a positive (and greater compared to dating apps) effect when it comes to facilitating long-distance unions.

### The selectivity of singles using dating apps

To examine the characteristics of dating app users in the pre-partnering stage, I here present the results of a supplementary analysis focusing on a sub-population of singles looking for a partner in Switzerland. I specifically ran three different analyses examining the potential self-selection of singles using swipe-based apps with regards to: 1) family formation intentions and family values; 2) psychological profile; and 3) in-person meeting opportunities and conditions.

The data for this analysis are drawn from wave 20 of the Swiss Household Panel (SHP), which is a stratified random sample of private households whose members represent the non-institutional resident population in Switzerland in 2018. The survey uniquely compliments the EFG data, which largely focused on couples, by including a measure on where singles search for partners in Switzerland. The SHP has been conducted annually since 1999, with refreshment samples (meant to ensure the continuing representativeness of the population in Switzerland) added in 2004 and 2013. The latest available wave (i.e., wave 20) was the first to collect information on the use of the Internet for partner search among single respondents between 18 and 60 years old. From the original sample of 13,751 participants, I excluded partnered respondents (*n* = 6,856), individuals younger than 18 or older than 60 (*n* = 3,865), household members who did not answer to the individual questionnaire (*n* = 1,744), and missing cases on relevant variables (*n* = 249). The analysis relied on a sample of 1,037 single respondents. Section 5 in S1 File provides details on measurements, the socio-demographic composition of the sample, as well as tables with the results of multivariate analyses.

First, findings in S5.2 Table in S1 File show that users of dating apps are not significantly different from non-users when it comes to gender values or religiosity. Nevertheless, singles seeking a partner through dating apps (both men and women, as an additional analysis including a gender interaction shows) are significantly more likely to mention wanting to have a child in the next two years. This effect is apparent for singles using dating websites, but the dating app effect is larger in magnitude.

As to psychological features (i.e., self-perception and sense of control, and most personality dimensions), there do not appear to be any significant differences between singles looking for a match through dating apps and those looking offline (S5.3 Table in S1 File). Dating app users however seem to be significantly more extroverted than singles not using virtual tools of mate selection.

Finally, the last set of analyses (S5.4 Table in S1 File) focusing on offline meeting opportunities and local marriage market conditions show that singles using dating websites are slightly less satisfied with personal relationships. This suggests that a potentially narrow social circle may push people into trying out dating platforms to meet partners. Dissatisfaction with personal relationships does not seem to be a selection mechanism for users of phone dating apps. What does appear as a more relevant aspect that predisposes people to use dating apps is limited time availability. Dating app users are significantly more likely than singles who do not use the Internet to search for a partner to mention being too exhausted after work to do what they would like.

## Discussion

Using nationally representative survey data from Switzerland, this study provided a rich overview of the demographic characteristics and union patterns of couples who met through dating apps in comparison to those who met offline or through other online contexts of partner selection. The evidence seems to suggest that non-residential couples formed through mobile dating have a greater interest in cohabitation than couples who met offline. The effect was not driven by socio-economic vulnerability, or a certain life course stage (it is valid even when controlling for education, employment status, and age), reflecting the increasingly universal appeal of non-marital coresidential unions across the Western world [13]. In the context of a consistently high divorce rate—with the exception of 2017 (38.7%), the divorce rate in Switzerland has been continually over 40% since 2002 [75] -, and knowing that stronger expectations to divorce predict a higher likelihood of remaining single or cohabiting first instead of marrying [76], one could envision that individuals using phone apps to find a partner cautiously opt for cohabitation over marriage as favored form of long-term union. Perhaps the pragmatic approach to finding a partner on dating apps [29] is also reflected in subsequent decisions regarding living arrangements. Nevertheless, the data also indicated that individuals in couples initiated through a dating app were not necessarily less interested in marriage than those in couples formed elsewhere. In a country where registered partnerships are not yet an option for opposite-sex couples, and where marriage is still seen as the ultimate partnership arrangement, equivalent to starting a family [12], couples who met on dating apps showing greater interest in cohabitation most likely see it as a stage preceding marriage. Results suggest that in Switzerland, the culture of dating apps promoting easy access to a large dating pool may lengthen the time people take to find the right marital partner, and encourage intermediary steps (i.e., cohabiting before marriage), but it may not ultimately deter marriage. Findings from the supplementary analysis examining the profile of dating app users in the pre-partnering stage (see S5.2 Table in S1 File) also suggest that singles relying on phone apps are not selectively less (or more) traditional in family values. Though app users score higher in extraversion (see S5.3 Table in S1 File), which has been positively linked to short-term mating [49], prevailing partnerships resulting from mobile dating did not appear short-term oriented. What is more, women who met their match on a dating app were more likely to mention wanting and intending to have a child in the near future than those who met their partner offline. This is less likely a result of more commitment-oriented individuals staying in unions and more likely a result of an initial selection mechanism, as the auxiliary analysis looking at singles indicated that users of dating apps had significantly stronger fertility intentions than non-users (S5.2 Table in S1 File).

This study additionally showed that relationship satisfaction or general subjective well-being did not differ between couples who met on dating apps and those who met in non-digital settings, mitigating concerns regarding the poor quality of unions formed in a partnership market often thought to only encourage frivolous image-based matching [29]. Nevertheless, supplementary analyses (S4.1 Fig in S1 File) suggested that within the sub-group of individuals in non-residential unions, those who met their partner on a dating app have a lower level of relationship satisfaction than those who met their partner via dating websites. This implies that among digital tools for dating, websites and their options for more refined searches may indeed represent a better way of finding a well-matched partner. This advantage is however absent when looking at more committed unions, most likely because of selective exists (i.e., unsatisfactory relationships not transitioning into cohabitation or marriage); in the long run, and as previously found [11], the context of meeting may have little effect on partnership quality.

The data furthermore revealed that, as expected due to extendible search options, the use of smartphone applications for dating facilitates relationships between geographically distant partners. Whereas on dating websites people seemingly need to search wider and more often end up in long-distance non-residential unions, dating apps, by affording greater spatial mobility and access to geographically adjacent as well as more distant spaces, facilitate relationships between individuals living both moderate and long distances from each other. It could be argued that given the costly nature of long-distance unions [64], couples started on dating apps having a more pronounced wish to cohabit may be related to distance, but the effect of meeting context on cohabiting intentions is robust to the inclusion of geographical exogamy, as additional analyses (not shown) revealed. In light of the lower likelihood of marriage among long-distance couples [77] however, couples formed on dating apps may ultimately differ in the probability of transitioning into marriage rather than the intention to marry.

Another finding is that partners who met through dating apps were more educationally exogamous than those who met elsewhere, particularly those who met through local networks of friends or associations, at school or at work. Especially for highly educated women marrying later in life and investing in professional careers (selectivity results show that app users are significantly more likely to experience work-life conflict, see S5.4 Table in S1 File), dating apps may effectively replace local dating pools and schools as key marriage markets [78]. It was also found that partnerships initiated on dating apps are not more exogamous on origin. Even though previous research identified a link between meeting online (through dating apps and websites combined) and exogamy on race in the U.S. [10], in Switzerland, it is other online meeting spaces such as social networks that encourage more cultural mixing. For immigrants, swipe-based apps are in fact a less appealing option for partner search to begin with (S5.1 Table in S1 File). Finally, results showed that couples who met on dating websites (but not on dating apps) were closer in age than those who met in conventional ways. Likely a result of a browsing interface that allows for sorting along age, this finding aligns with previous work identifying the same pattern for couples who met through online dating (in general) in the U.S. [10]. Dating apps also include information on partners’ age in the foreground, but probably given a larger supply of candidates, they discourage age homogamy especially among younger adults (i.e., under 30). Nevertheless, as predicted, the increase in couples with a big age gap between partners is in fact an increase in age-hypergamous unions, perpetuating stereotypical gendered pairings of men dating younger women. Future work should investigate this further, as well as explore the role of dating apps in changing assortative mating along other dimensions, such as political orientation, religion, or social origin (measures absent in the data set at hand).

In addition to these extensions, attention must also be given to actual transitions into cohabitation and marriage, as well as the question of longevity. An important limitation of this study is that by means of using cross-sectional data, it only examined a snapshot of already established couples. Even though the data set included partnerships with a lower level of commitment than marriage (i.e., non-residential unions), it could not capture casual encounters or short-term-oriented connections that never formalize. Therefore, the hypothesis of apps users transitioning less into actual partnerships given the overload of choice or the objectification of potential mates is yet to be refuted. Future studies should track the full-range of partnering choices (from casual dating, hook-ups, to long-term relationships) that singles experience based on the different dating strategies they resort to. Retracing partnership trajectories in more detail could also identify how common it is that connections initiated through apps start as flings but eventually develop into more committed unions. It may be that despite their reputation and the presumed superficiality of swipe-based courtship, dating apps are representative of a modern dating culture where relationships that begin as hook-ups or short-term flings are not thereby excluded from developing into meaningful long-term connections [2, 7, 79]. A within-subject design that follows the same set of individuals in both the pre- and post-partnering phase could also allow for analyses that minimize the risk of endogeneity and unobserved selection bias. Furthermore, though the volume of the data used in this study was sufficient to warrant a distinction between different online meeting contexts, the sub-sample of respondents who met their partner through dating apps for instance was still rather modest. Future research should re-visit these questions with much larger samples. Finally, I invite future work to replicate this study in other national contexts. Current results may be generalized to countries in which marriage (with children) is still normatively and institutionally endorsed, and where individuals in search for partners online can only take advantage of a less socially constraining mate selection context by showing more interest in (pre-marital) cohabitation. It may be that in other less conservative countries or in contexts where hook-ups are already engrained in partnering culture, like the U.S. [16, 80], users of swipe-based apps are more susceptible to the casual dating ethos of swipe-based apps, and hence more frequently engage in uncommitted sexual encounters, or form couples with looser family formation intentions. Furthermore, data from countries with a more rapid expansion of women’s education [81, 82], and thus a greater over-supply of high-educated women in search of partners, may reveal an even stronger effect of dating apps on educational exogamy. Focusing on other countries could also reveal whether online social networks pervasively encourage more unions between natives and people with different immigrant background, and whether the hook-up-centric reputation of dating apps deters immigrants looking for more traditional arrangements.

Though it is still early to draw conclusions about the long-term impact of this unique way of selecting and matching with partners, this study provides a first indication that compared to couples formed through other settings, those initiated on dating apps do not shy away from long-term commitment, nor experience low-quality connections. Lastly, results showed that unions started on dating apps exhibit greater exogamy on certain attributes (geography, education, and to a limited extent, age) but not on others (e.g., origin group).

## Supporting information

S1 File (DOCX)
