# Supplementary material for: The demography of swiping right. An overview of couples who met through dating apps in Switzerland
Source: PLoS One. 2020 Dec 30;15(12):e0243733. doi: 10.1371/journal.pone.0243733 (PMC7773176; doi:10.1371/journal.pone.0243733)
Supplement: S1 File — (DOCX) [file pone.0243733.s001.docx]

*The Demography of Swiping Right.*

*An Overview of Couples Who Met through Dating Apps in Switzerland*

**Online Supplement**

**Table of Contents**

[1. Descriptive Statistics 3](#_Toc57307549)

[2. How Heterosexual Couples Met in Switzerland – Unadjusted Percentages 4](#_Toc57307550)

[3. Tables with Full Model Coefficients 5](#_Toc57307551)

[4. Additional Analyses 8](#_Toc57307552)

[5. The Selectivity of Singles Using Dating Apps 12](#_Toc57307553)

[5.1. Measurements & Descriptive Statistics 12](#_Toc57307554)

[5.2. Results 15](#_Toc57307555)

[6. Replication of Analyses on Recently Formed Couples 18](#_Toc57307556)

[7. References 22](#_Toc57307557)

## Descriptive Statistics

Table S1 provides an overview of the sample used in the analyses reported in the main text, by meeting context. Individuals who met their match through an app are the youngest (e.g., 84.3% are under 40, compared to 71.6% among those who met their partner offline or only 39.1% among those who met through dating websites). Furthermore, they are less likely to be married, but more likely to be in a non-residential union. Compared to respondents who met their partner offline, those who used dating apps are also more often part of a same-sex union (though the largest number of same-sex unions started through other online venues), less often highly educated, and much less likely to have biological children in general or with their current partner. Individuals who met their partner via a dating website or app less often have a migration background. Finally, relationships started via phone apps are the shortest (i.e., 2.32 years on average compared to 4.96 among couples who met offline).

**Table S1:** **Socio-demographic composition of sample, by meeting context** (*N = 3,245*)

|  | Offline | Dating  app | Dating website | Other online | sig. |
| --- | --- | --- | --- | --- | --- |
|  | %/ Mean (Standard Deviation) | | | |  |
| Female | 49.0 | 52.6 | 48.8 | 51.9 |  |
| Same-sex couple | 1.2 | 2.8 | 2.7 | 7.2 | *** |
| Type of union |  |  |  |  | * |
| Marriage | 29.7 | 11.2 | 27.0 | 32.8 |  |
| Cohabiting | 32.5 | 30.0 | 34.1 | 30.5 |  |
| Non-residential | 37.8 | 58.8 | 38.9 | 36.8 |  |
| Age |  |  |  |  | *** |
| 18-29 | 36.6 | 50.2 | 5.6 | 53.7 |  |
| 30-39 | 35.0 | 34.1 | 33.5 | 22.6 |  |
| 40+ | 28.5 | 15.7 | 60.9 | 23.8 |  |
| Tertiary education | 32.3 | 20.6 | 34.5 | 23.4 | * |
| Employed | 87.1 | 87.6 | 87.3 | 91.4 |  |
| Previously married | 25.9 | 25.0 | 38.7 | 22.6 | * |
| Prior cohabitation | 37.8 | 37.7 | 65.2 | 33.4 | *** |
| Biological children | 37.2 | 18.5 | 56.2 | 21.6 | *** |
| Common children | 23.0 | 9.9 | 18.8 | 16.5 | ** |
| Religious | 45.9 | 40.7 | 51.7 | 41.2 |  |
| Migration background | 40.6 | 25.0 | 31.3 | 46.6 | * |
| Region |  |  |  |  |  |
| Densely populated | 32.2 | 30.0 | 26.8 | 23.8 |  |
| Moderately urbanized | 50.5 | 52.6 | 55.5 | 62.0 |  |
| Sparsely populated | 17.4 | 17.4 | 17.8 | 14.3 |  |
| Work-life conflict (range: 3-20) | 9.16 (0.08) | 9.55 (0.35) | 9.22 (0.30) | 9.18 (0.29) |  |
| Poor health | 12.6 | 19.6 | 19.6 | 20.0 | * |
| Partnership duration (range: 0-10) | 4.96 (0.08) | 2.32 (0.22) | 4.72 (0.24) | 4.69 (0.39) | *** |
| *N (unweighted)* | *2,752* | *104* | *264* | *125* |  |

Source: EFG 2018.

Note: Weighted data by wecritpers. † *p* < .10; * *p* < 0.05; ** *p* < 0.01; *** *p* < 0.001.

## How Heterosexual Couples Met in Switzerland – Unadjusted Percentages

**Table S2:** **How heterosexual couples met in Switzerland, by year of meeting (1995-2018) - row %**

|  | Friends | Family | At school or work | Bar or restaurant | Hobby, association | Other offline | Dating website | Dating app | Other online | Online - Total | *Total* |
| --- | --- | --- | --- | --- | --- | --- | --- | --- | --- | --- | --- |
| 1995 | 31.10 | 5.89 | 27.06 | 19.52 | 5.24 | 11.08 | 0.11 | 0.00 | 0.00 | 0.11 | *251* |
| 1996 | 30.52 | 2.15 | 22.49 | 20.57 | 14.14 | 9.82 | 0.31 | 0.00 | 0.00 | 0.31 | *257* |
| 1997 | 30.63 | 3.37 | 28.46 | 15.61 | 9.56 | 10.58 | 1.40 | 0.00 | 0.39 | 1.79 | *255* |
| 1998 | 29.83 | 4.90 | 25.84 | 15.11 | 13.76 | 10.28 | 0.00 | 0.00 | 0.28 | 0.28 | *364* |
| 1999 | 32.37 | 4.10 | 30.28 | 13.96 | 7.60 | 7.14 | 2.17 | 0.00 | 2.38 | 4.55 | *304* |
| 2000 | 30.94 | 3.61 | 25.97 | 20.42 | 8.11 | 7.59 | 2.09 | 0.00 | 1.28 | 3.37 | *415* |
| 2001 | 36.47 | 2.76 | 21.08 | 15.22 | 10.56 | 10.54 | 2.18 | 0.00 | 1.18 | 3.36 | *367* |
| 2002 | 34.30 | 3.86 | 22.42 | 17.69 | 5.90 | 10.63 | 3.51 | 0.00 | 1.68 | 5.19 | *361* |
| 2003 | 27.60 | 4.23 | 22.44 | 20.28 | 8.23 | 10.32 | 3.60 | 0.00 | 3.31 | 6.90 | *399* |
| 2004 | 28.53 | 6.73 | 18.42 | 17.91 | 5.79 | 11.14 | 5.93 | 0.00 | 5.56 | 11.49 | *314* |
| 2005 | 25.75 | 6.70 | 27.85 | 12.96 | 9.48 | 9.78 | 4.94 | 0.00 | 2.54 | 7.48 | *333* |
| 2006 | 21.98 | 4.65 | 23.84 | 18.61 | 5.49 | 12.87 | 6.14 | 0.00 | 6.42 | 12.56 | *352* |
| 2007 | 27.84 | 5.11 | 23.69 | 11.19 | 11.54 | 11.81 | 5.48 | 0.00 | 3.34 | 8.82 | *353* |
| 2008 | 27.70 | 5.28 | 21.78 | 17.51 | 5.76 | 9.32 | 5.91 | 0.00 | 6.73 | 12.64 | *418* |
| 2009 | 32.98 | 6.29 | 19.51 | 17.77 | 6.14 | 6.16 | 8.08 | 0.43 | 2.65 | 11.17 | *301* |
| 2010 | 26.68 | 5.21 | 20.44 | 16.89 | 9.18 | 7.23 | 5.56 | 0.41 | 8.40 | 14.37 | *308* |
| 2011 | 36.42 | 3.42 | 20.99 | 19.20 | 4.16 | 7.00 | 7.52 | 0.43 | 0.86 | 8.82 | *300* |
| 2012 | 26.96 | 4.28 | 22.35 | 18.47 | 4.57 | 8.71 | 4.99 | 3.54 | 6.14 | 14.67 | *301* |
| 2013 | 26.09 | 2.02 | 22.47 | 13.73 | 6.68 | 15.02 | 9.19 | 1.86 | 2.93 | 13.98 | *319* |
| 2014 | 26.29 | 3.11 | 18.42 | 16.50 | 6.27 | 12.85 | 8.09 | 3.95 | 4.53 | 16.57 | *293* |
| 2015 | 27.10 | 3.52 | 20.04 | 12.35 | 11.34 | 8.88 | 9.72 | 2.73 | 4.33 | 16.77 | *267* |
| 2016 | 27.41 | 2.37 | 17.47 | 18.32 | 9.25 | 7.53 | 5.20 | 7.81 | 4.64 | 17.65 | *276* |
| 2017-18 | 26.91 | 1.63 | 18.15 | 14.91 | 7.57 | 5.91 | 7.80 | 10.33 | 6.81 | 24.93 | *413* |
| *N* (unweighted) | *2243* | *325* | *1750* | *1246* | *628* | *656* | *385* | *100* | *188* | *673* | *7,521* |

Source: EFG 2018.

Note: Weighted data by wecritpers.

## Tables with Full Model Coefficients

**Table S3.1:** **Logistic regression coefficients predicting family formation intentions by meeting context**

|  | Marital Intentions | Cohabiting Intentions | Fertility Desire | Fertility Intentions |
| --- | --- | --- | --- | --- |
| Meeting context (ref.: offline) |  |  |  |  |
| Dating app | 0.034 | 1.353** | 0.392 | 0.481 |
| Dating website | −0.100 | 0.505 | −0.143 | 0.259 |
| Other online | 0.581 | 0.758 | 0.187 | 0.559† |
| Female | −0.117 | −0.769*** | −0.439** | −0.207 |
| Same-sex couple |  | −1.265† | −1.910*** | −1.520** |
| Type of union (ref. : marriage) |  |  |  |  |
| Cohabiting | ref. |  | 0.323 | −0.089 |
| Non-residential | −1.275*** |  | −0.031 | −1.098*** |
| Age (ref.: 18-29) |  |  |  |  |
| 30-39 | 0.040 | −0.227 | −1.641*** | 0.664*** |
| 40+ | −0.713* | −2.207*** | −3.831*** | −1.076*** |
| Tertiary education | 0.279 | 0.173 | 0.436** | 0.225† |
| Employed | 0.478† | 0.391 | 0.435* | 0.419† |
| Previously married | −0.145 | −0.343 | −0.809*** | −0.263 |
| Prior cohabitation | −0.190 | 0.871** | −0.364* | −0.030 |
| Biological children | −0.648** | −0.006 |  |  |
| Common children |  |  | −0.847*** | −0.351* |
| Religious | 0.640*** | 0.234 | 0.489*** | 0.319* |
| Migration background | 0.811*** | 0.501* | 0.359* | 0.168 |
| Work-life conflict | 0.032 | 0.024 | −0.004 | −0.0002 |
| Poor health | −0.300 | −0.068 | −0.485* | −0.555** |
| Relationship satisfaction | 0.240*** | 0.257*** | 0.101* | 0.053 |
| Partnership duration | 0.056 | −0.003 | −0.133*** | −0.043 |
| Constant | −3.658*** | −1.683** | 2.303*** | −0.653 |
| Pseudo R-squared | 0.166 | 0.193 | 0.361 | 0.122 |
| *N* | *1,908* | *928* | *2,651* | *2,649* |

Source: EFG 2018.

Note: Weighted data by wecritpers. † *p* < .10; * *p* < 0.05; ** *p* < 0.01; *** *p* < 0.001.

**Table S3.2: OLS regression coefficients predicting relationship and life satisfaction by meeting context**

|  | Relationship Satisfaction | Life  Satisfaction |
| --- | --- | --- |
| Meeting context (ref.: offline) |  |  |
| Dating app | −0.171 | −0.228 |
| Dating website | 0.190† | 0.080 |
| Other online | −0.303 | −0.462* |
| Female | 0.022 | −0.029 |
| Same-sex couple | −0.113 | 0.099 |
| Type of union (ref. : marriage) |  |  |
| Cohabiting | −0.293** | −0.302* |
| Non-residential | −0.815*** | −0.651*** |
| Age (ref.: 18-29) |  |  |
| 30-39 | −0.396*** | −0.251* |
| 40+ | −0.407** | −0.234† |
| Tertiary education | −0.150* | 0.077 |
| Employed | 0.393*** | 0.848*** |
| Previously married | 0.108 | 0.230* |
| Prior cohabitation | −0.154 | 0.026 |
| Biological children | −0.278** | −0.021 |
| Religious | 0.054 | 0.143† |
| Migration background | −0.008 | −0.346*** |
| Work-life conflict | −0.091*** | −0.147*** |
| Poor health | −0.336** | −1.336*** |
| Partnership duration | −0.035* | −0.023 |
| Constant | 10.064*** | 9.155*** |
| R-squared | 0.088 | 0.203 |
| *N* | *3,206* | *3,210* |

Source: EFG 2018.

Note: Weighted data by wecritpers. † *p* < .10; * *p* < 0.05; ** *p* < 0.01; *** *p* < 0.001.

**Table S3.3:** **Logistic and multinomial logistic regression coefficients predicting exogamy by meeting context**

|  | Educational Exogamy | Exogamy on Origin  among Natives | Exogamy on Origin  among Migrants | Age Difference  (Ref.: Age Hypergamy) | | Geographical Exogamy  (Ref.: Short Distance) | |
| --- | --- | --- | --- | --- | --- | --- | --- |
|  | Logit | Logit | Logit | Multinomial Logit | | Multinomial Logit | |
|  |  |  |  | Age Homogamy | Age Hypogamy | Moderate Distance | Long  Distance |
| Meeting context (ref.: offline) |  |  |  |  |  |  |  |
| Dating app | 0.650* | 0.228 | 0.641 | −0.327 | −0.143 | 0.968* | 1.062* |
| Dating website | 0.010 | 0.237 | 0.347 | 0.511** | −0.190 | 0.571 | 1.394** |
| Other online | −0.260 | 1.314*** | −0.202 | 0.061 | −0.398 | −0.120 | 0.690 |
| Female | 0.224† | −0.183 | 0.397* | −0.135 | −0.099 | −0.031 | 0.046 |
| Same-sex couple | −0.316 | 1.449** | 0.857† |  |  | −0.040 | 1.318† |
| Type of union (ref. : marriage) |  |  |  |  |  |  |  |
| Cohabiting | −0.078 | −0.874*** | 0.538* | 0.111 | −0.306 |  |  |
| Non-residential | 0.171 | −1.070*** | 0.806** | 0.252 | −0.160 |  |  |
| Age (ref.: 18-29) |  |  |  |  |  |  |  |
| 30-39 | 0.064 | 0.477* | −0.009 | −0.662*** | 0.416 | 0.088 | 0.346 |
| 40+ | 0.091 | 0.929*** | −0.072 | −1.229*** | 0.465 | 0.633 | 0.469 |
| Tertiary education | 0.796*** | 0.319* | −0.196 | 0.077 | −0.197 | 0.281 | 1.061*** |
| Employed | 0.038 | −0.169 | 0.065 | −0.172 | −0.234 | 0.265 | −0.688* |
| Previously married | −0.001 | 0.408* | −0.005 | −0.149 | −0.093 | −0.472 | 0.064 |
| Prior cohabitation | 0.312* | −0.031 | 0.381† | −0.093 | 0.287 | 0.142 | 0.208 |
| Biological children | −0.160 | −0.239 | −0.251 | −0.072 | −0.207 | −0.368 | −0.582† |
| Religious | 0.093 | 0.203 | −0.255 | −0.025 | 0.129 | 0.278 | 0.641** |
| Migration background | 0.052 |  |  | −0.147 | −0.173 | 0.033 | 0.202 |
| Region (ref.: densely populated) |  |  |  |  |  |  |  |
| Moderately urbanized | 0.054 | −0.507** | 0.476* | −0.020 | −0.027 | 0.211 | 0.114 |
| Sparsely populated | 0.159 | −0.662** | 0.622* | 0.161 | −0.060 | −0.151 | 0.086 |
| Work-life conflict | 0.019 | 0.029 | −0.040 | 0.026 | 0.015 | 0.073† | 0.111** |
| Partnership duration | 0.015 | −0.107** | 0.019 | 0.025 | −0.043 | −0.154** | 0.010 |
| Constant | −2.065*** | −0.540 | −1.116* | 0.227 | −1.298** | −1.569** | −2.207*** |
| (Pseudo) R-squared | 0.036 | 0.078 | 0.064 | 0.048 | | 0.084 | |
| *N* | *2,801* | *1,955* | *1,240* | *3,156* | | *925* | |

Source: EFG 2018.

Note: Weighted data by wecritpers. † *p* < .10; * *p* < 0.05; ** *p* < 0.01; *** *p* < 0.001.

## Additional Analyses

**Table S4:** **Logistic regression coefficients predicting educational exogamy by meeting context (detailed categorization)**

|  | Educational Exogamy | |
| --- | --- | --- |
|  | Coeff. (SE) | |
| Meeting context (ref.: dating app) |  |  |
| Through friends or acquaintances | −0.720* | (0.329) |
| Through family | −0.503 | (0.438) |
| At school, through studies, at work | −0.912** | (0.341) |
| In a bar, a disco, a concert, a neighborhood party, … | −0.485 | (0.347) |
| Through a hobby, association, sports’ club | −0.756* | (0.378) |
| Dating website | −0.641† | (0.370) |
| Other online | −0.918* | (0.416) |
| Other | −0.284 | (0.353) |
| Pseudo R-squared | 0.0414 | |
| *N* | *2,801* | |

Source: EFG 2018.

Note: Weighted data by wecritpers. The model controls for: gender, same-sex couple, type of union, age, tertiary education, whether employed, previously married, prior cohabitation, biological children, religiosity, migration background, type of residential region, work-life conflict, and partnership duration.

† *p* < 0.10; * *p* < 0.05, ** *p* < 0.01.


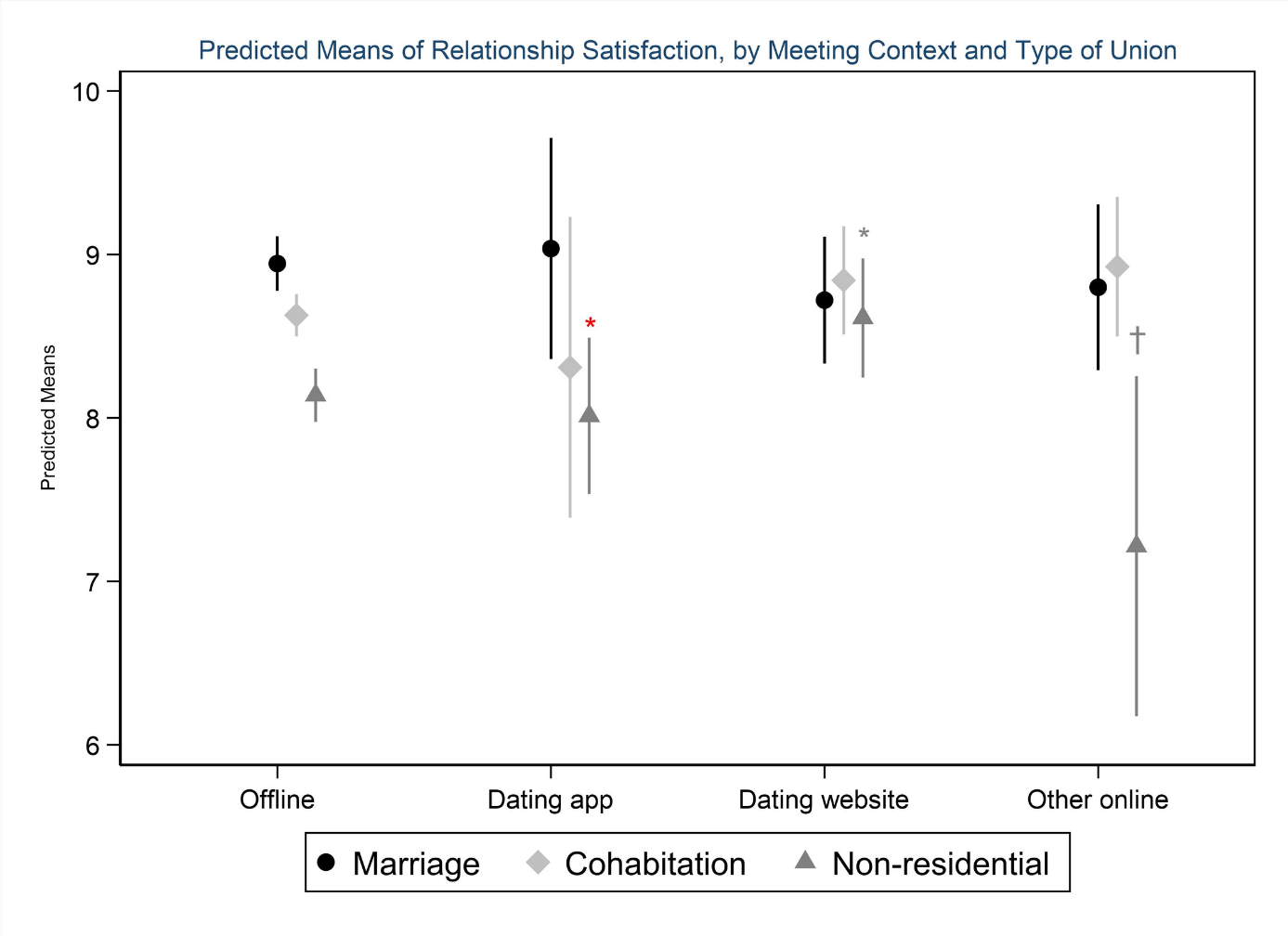


**Figure S4.1:** Predicted means of relationship satisfaction, by meeting context and type of union, 95% CI

*Note*: Based on an OLS model including interactions between meeting context and type of union, controlling for gender, same-sex couple, age, tertiary education, whether employed, previously married, prior cohabitation, biological children, religiosity, migration background, work-life conflict, poor health, and partnership duration.

Stars (in dark grey) indicate means significantly different compared to the offline category: † *p* < 0.10; * *p* < .05. Stars (in red) indicates a significant contrast between the dating app and dating website category: * *p* < .05.


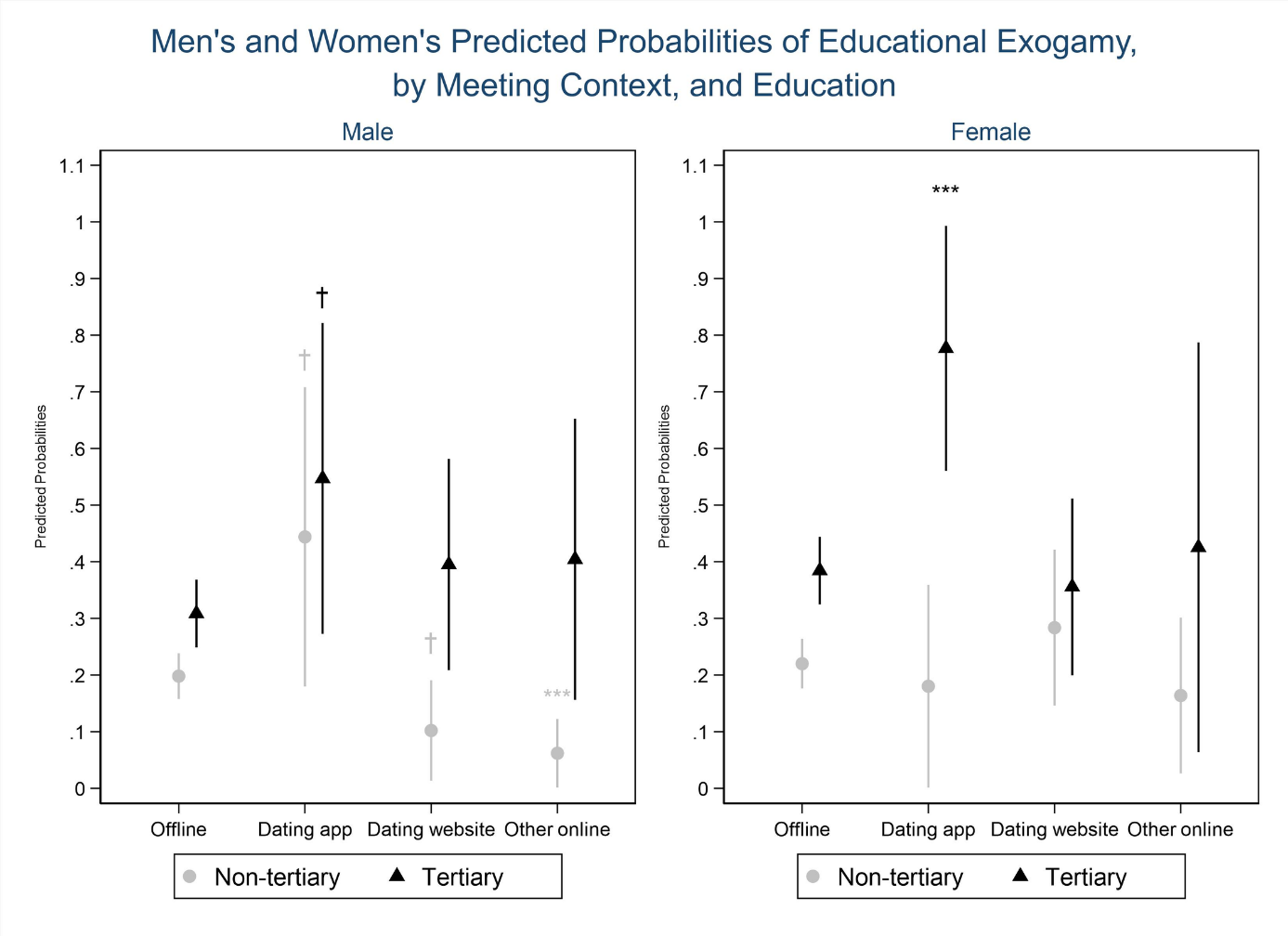


**Figure S4.2:** Predicted probabilities of educational exogamy, by meeting context, education and gender, 95% CI

*Note*: Based on a logistic regression model including interactions between meeting context, education, and gender, controlling for same-sex couple, type of union, age, whether employed, previously married, prior cohabitation, biological children, religiosity, migration background, type of residential region, work-life conflict, and partnership duration. Stars indicate probabilities significantly different compared to the offline category: † *p* < 0.10; *** *p* < .001.


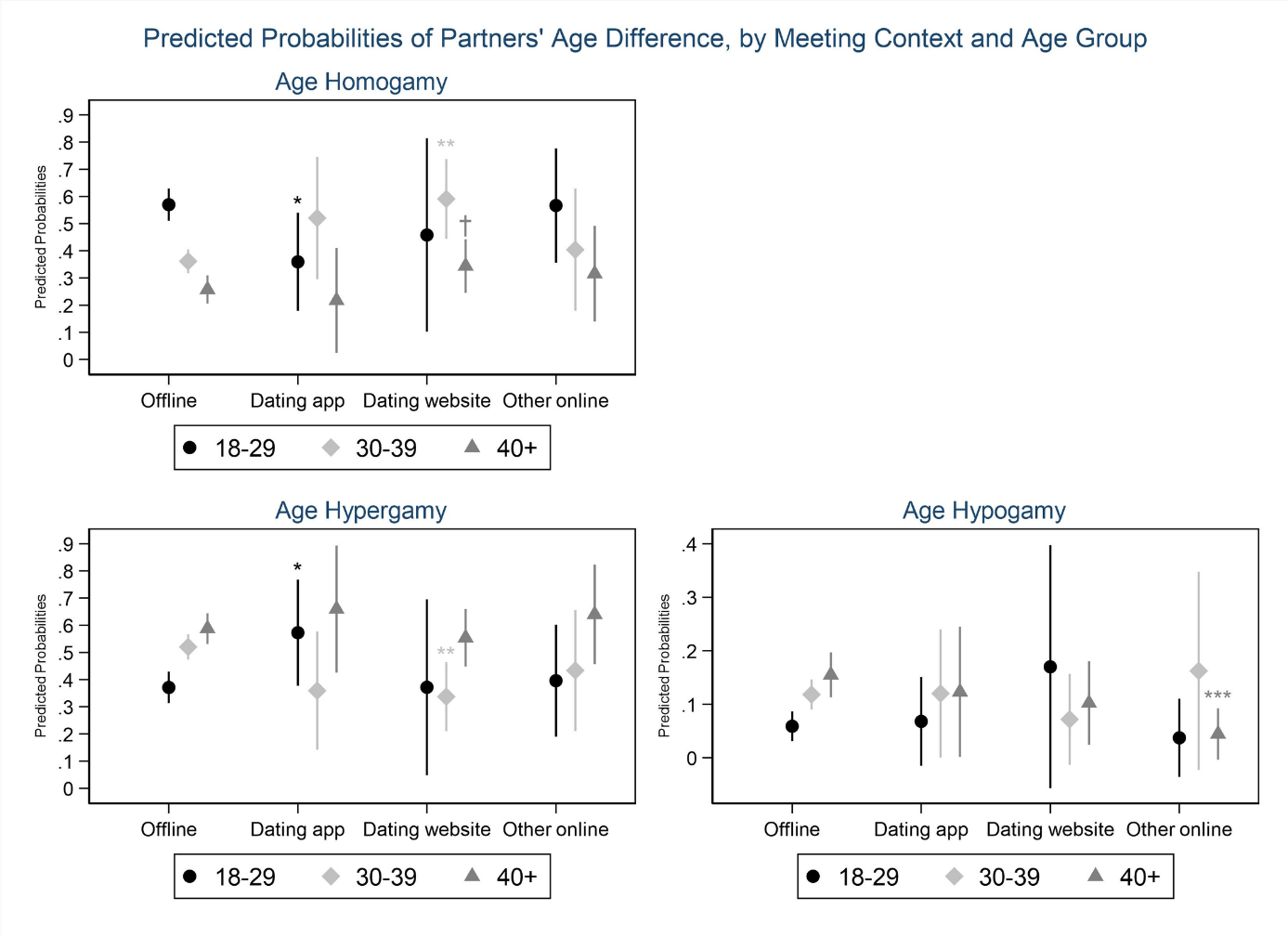


**Figure S4.3:** Predicted probabilities of age hypergamy, age homogamy, and age hypogamy, by meeting context and age group, 95% CI

*Note*: Based on multinomial logistic regression models including interactions between meeting context and age group, controlling for gender, type of union, age, whether employed, previously married, prior cohabitation, biological children, religiosity, migration background, type of residential region, work-life conflict, and partnership duration. Stars indicate probabilities significantly different compared to the offline category: † *p* < 0.10; * *p* < .05; ** *p* < .01; *** *p* < .001.

## The Selectivity of Singles Using Dating Apps

#### Measurements & Descriptive Statistics

The information on the digital tools that singles use to search for partners was obtained via the item “Which Internet service have you used most to look for a partner?”, provided that respondents answered “yes” to a preceding question that asked “In the past year, have you used the internet to look for a partner?”. The five options that the respondents could choose from included: 1) dating websites (e.g. eDarling, Parship, Swissfriends, OkCupid), 2) phone dating apps (e.g. Tinder, Lovoo, Grindr, Zoosk); 3) social network sites (e.g. Facebook, Instagram, Twitter, Meetup); 4) chat rooms (e.g. Swisstalk, Citychat, Chatroulette); and 5) other. To construct a broad measure of where people look for partners, I combined information on both variables and created the following categorization of partner search context: 1) offline (did not use the Internet); 2) dating apps; 3) dating platforms; and 4) other online venues (combining social network sites, chat rooms, and other).

Family formation intentions are captured via an item measuring fertility plans. This item asks “Do you intend to have a child in the next 24 months?”, with answer options “yes” and “no”. In the prompt, it was clarified that in cases of current pregnancy, the answer should not count the child couples were currently expecting. The question was asked to male respondents between 18 and 50 years old, and female respondents between 18 and 45 years old, who had provided a non-negative answer to a previous question asking “All in all, how many children would you like to have?”. Items measuring marital or cohabiting preferences (for un-partnered respondents) are missing.

In the absence of measures of marriage attitudes, I scrutinized other types of values that often relate to traditional versus progressive orientations towards family, namely gender values. Attitudes regarding gender roles within a family were captured via two items measured on a 0 (“completely disagree”)-10 (“completely agree”) scale: “To have a job is the best guarantee for a woman as for a man to be independent”, and “A pre-school child suffers, if his or her mother works for pay”. The SHP module on gender role attitudes was last included in wave 19, so the measures are essentially lagged by one year. Furthermore, as described earlier, religiosity is also used as proxy for traditional family attitudes. This concept, included in wave 20, was measured through the question “All in all, how religious would you consider yourself to be?”, with answer possibilities: “not at all”, “not very much”, “moderately”, “quite”, and “very”. Respondents who chose either one of last three categories were considered religious.

To examine the psychological profile of dating app users, I specifically looked at a scale of self-perception and control, and personality dimensions. The former (included in wave 20) was measured via a mean score of twelve items (Cronbach alpha 0.791) on a 0 (“completely disagree”) to 10 (“completely agree”) scale. The items either refer to self-efficacy toward one’s environment (inability to make plans because of unpredictability; little influence on life events; ability to overcome unexpected problems; ability to choose between two possibilities), self-esteem (feeling of uselessness; feeling of self-satisfaction), personal mastery (doing everything I set my mind to; finding a way to succeed; what I want is in my hands; what will happen depends on me), or perceived constraints (others determine what I can do; feeling of being pushed around in life). To compute the mean score, certain items that were initially framed in positive valence (e.g., ability to overcome unexpected problems) were reversed. The resulting score ranged from 2.08 to 10, with higher values indicating negative self-perception and low control over one’s circumstances. Personality dimensions were only included in wave 17 (2015) and were based on the 15-item Big Five Inventory-Short Version (BFI-15; [1]). This version of the Big Five includes three items per personality trait. Each item goes from zero “disagree strongly” to ten “agree strongly” and measures how an individual positions himself relative to a list of 15 statements.

Finally, in-person meeting opportunities and conditions were captured via four items, all measured on 0 “not at all” to 10 “completely/ strongly” scales. Satisfaction with personal relationships (“How satisfied are you with your personal, social and family relationships?”) was intended to indicate the quality of offline networks of friends. To capture the amount of free time available to search for partners in offline circles, the other three items referred to work-life conflict (“Exhausted after work to do what you would like”, “How difficult to disconnect from work when the work day is over”), and level of satisfaction with free time (“How satisfied are you with the amount of free time you have?”).

Similar to the models reported in the main text, the current set of analyses included several controls, based on similar schemes of operationalization, such as gender, age category, tertiary education, being employed, previous marital experience, biological children, migration background, and poor health. The summary statistics provided in Table S5.1 reveal that in several ways, singles using dating apps have a similar socio-demographic profile compared to partnered individuals who met their match using mobile dating (e.g., younger, less likely to have a migration background, less likely to have children). We also see that women are under-represented on dating apps, but over-represented among singles searching for a partner on dating websites. The over-representation of individuals with tertiary education among singles searching for a match on dating apps but not among those who already found a partner, indicates a lower probability of actually finding a match for well-educated singles using dating apps.

**Table S5.1:** **Socio-demographic composition of sample, by search context** (*N = 1,189*)

|  | Offline | Dating  app | Dating website | Other online | sig. |
| --- | --- | --- | --- | --- | --- |
|  | %/ Mean (Standard Deviation) | | | |  |
| Female | 47.3 | 30.2 | 64.2 | 32.1 | ** |
| Age |  |  |  |  | *** |
| 18-29 | 43.5 | 58.0 | 7.9 | 50.2 |  |
| 30-39 | 16.0 | 20.8 | 31.0 | 16.2 |  |
| 40+ | 40.5 | 21.2 | 61.0 | 33.6 |  |
| Tertiary education | 21.6 | 29.8 | 37.3 | 10.4 | † |
| Employed | 76.8 | 85.3 | 96.4 | 85.5 | * |
| Previously married | 18.5 | 13.0 | 46.8 | 11.8 | *** |
| Biological children | 23.2 | 15.2 | 39.5 | 7.5 | * |
| Migration background | 28.1 | 17.8 | 44.2 | 0.0 | * |
| Poor health | 15.5 | 6.3 | 27.7 | 13.5 | * |
| *N (unweighted)* | *819* | *125* | *71* | *22* |  |

*Source*: SHP 2018 (wave 20).

*Note*: Weighted data by wicss. † *p* < 0.10; * *p* < 0.05; ** *p* < 0.01; *** *p* < 0.001.

#### Results

**Table S5.2:** **Regression coefficients predicting singles’ family formation intentions and values, by search context**

|  | Fertility Intentions | Gender Values: Job preserves independence | Gender Values: Child suffers with working mother | Religiosity |
| --- | --- | --- | --- | --- |
|  | Logit | OLS | OLS | Logit |
| Search context (ref.: offline) |  |  |  |  |
| Dating app | 2.478** | −0.049 | 0.139 | −0.520 |
| Dating website | 2.628** | 0.089 | 0.002 | −0.387 |
| Other online | 2.461† | 0.209 | 1.363** | −0.046 |
| Female | −0.527 | 0.495** | −1.059*** | 0.133 |
| Age (ref.: 18-29) |  |  |  |  |
| 30-39 | 4.011*** | 0.182 | −0.491 | 0.009 |
| 40+ | 3.114* | 0.898*** | −0.067 | 0.814** |
| Tertiary education | 0.473 | 0.217 | −1.369*** | −0.384 |
| Employed | 0.112 | −0.270 | −0.052 | −0.401 |
| Previously married | 0.461 | 0.034 | 0.152 | 0.300 |
| Biological children |  | −0.045 | −0.456 | −0.326 |
| Migration background | 0.938 | 0.066 | 1.005** | 0.232 |
| Poor health | 0.070 | −0.365 | 0.627† | 0.183 |
| Constant | −8.031*** | 7.671*** | 5.615*** | −0.991*** |
| R-squared |  | 0.069 | 0.126 |  |
| *N* | *855* | *878* | *869* | *1,037* |

*Source*: SHP 2018 (wave 20).

*Note*: Weighted data by wicss. † *p* < 0.10; * *p* < 0.05; ** *p* < 0.01; *** *p* < 0.001.

**Table S5.3:** **OLS regression coefficients predicting singles’ psychological profile, by search context**

|  | Self-perception and Control | Neuroticism | Extraversion | Conscientiousness | Agreeableness | Openness |
| --- | --- | --- | --- | --- | --- | --- |
| Search context (ref.: offline) |  |  |  |  |  |  |
| Dating app | 0.038 | 0.010 | 0.358* | −0.098 | −0.278 | −0.266 |
| Dating website | 0.099 | 0.069 | 0.254 | 0.306 | 0.112 | 0.400 |
| Other online | 0.017 | 0.264 | 0.542† | 0.008 | 0.207 | −0.112 |
| Female | 0.122 | 0.437** | 0.076 | 0.412** | 0.374* | 0.398* |
| Age (ref.: 18-29) |  |  |  |  |  |  |
| 30-39 | −0.085 | 0.448 | 0.189 | 0.929*** | −0.125 | 0.165 |
| 40+ | −0.227* | −0.091 | −0.198 | 1.020*** | −0.255 | 0.278 |
| Tertiary education | −0.024 | −0.163 | −0.215 | −0.190 | 0.171 | −0.244 |
| Employed | 0.100 | −0.349† | 0.183 | 0.141 | −0.039 | −0.161 |
| Previously married | 0.019 | 0.241 | 0.253 | −0.201 | −0.120 | −0.187 |
| Biological children | 0.363** | −0.196 | 0.017 | 0.120 | 0.345 | −0.307 |
| Migration background | −0.449*** | 0.450 | 0.581* | −0.004 | −0.621 | −0.073 |
| Poor health | −1.091*** | 0.580* | −0.713** | −0.628** | 0.207 | −0.252 |
| Constant | 7.049*** | 4.374*** | 4.917*** | 6.356*** | 7.016*** | 6.436*** |
| R-squared | 0.171 | 0.065 | 0.075 | 0.195 | 0.060 | 0.039 |
| *N* | *1,037* | *580* | *580* | *580* | *580* | *580* |

*Source*: SHP 2018 (wave 20).

*Note*: Weighted data by wicss. † *p* < 0.10; * *p* < 0.05; ** *p* < 0.01; *** *p* < 0.001.

**Table S5.4:** **OLS regression coefficients predicting singles’ offline opportunities, by search context**

|  | Satisfaction with Personal Relationships | Exhausted after work to do what you would like | How difficult to disconnect from work | Satisfaction with Free Time |
| --- | --- | --- | --- | --- |
| Search context (ref.: offline) |  |  |  |  |
| Dating app | −0.045 | 0.624* | −0.386 | −0.346 |
| Dating website | −0.465† | 0.415 | 0.176 | −0.004 |
| Other online | −0.028 | 0.450 | −0.241 | 0.655 |
| Female | 0.634*** | 0.461* | 0.208 | −0.071 |
| Age (ref.: 18-29) |  |  |  |  |
| 30-39 | −0.461* | 0.339 | 0.787* | −0.062 |
| 40+ | −0.518** | 0.720** | 1.198*** | 0.336 |
| Tertiary education | −0.305* | −0.355 | 0.569* | −0.127 |
| Employed | −0.004 | 0.000 | 0.000 | −1.076*** |
| Previously married | −0.047 | −0.361 | −0.483 | 0.031 |
| Biological children | 0.462† | −0.478 | −0.676* | 0.130 |
| Migration background | −0.131 | 1.005** | 0.006 | −0.596* |
| Poor health | −1.096*** | 1.791*** | 1.298*** | −1.197*** |
| Constant | 7.880*** | 3.833*** | 2.432*** | 7.812*** |
| R-squared | 0.136 | 0.124 | 0.095 | 0.080 |
| *N* | *1,037* | *844* | *844* | *1,036* |

*Source*: SHP 2018 (wave 20).

*Note*: Weighted data by wicss. † *p* < 0.10; * *p* < 0.05; ** *p* < 0.01; *** *p* < 0.001.

## Replication of Analyses on Recently Formed Couples

**Table S6.1:** **Logistic regression coefficients predicting family formation intentions by meeting context – among recently formed couples**

|  | Marital Intentions | Cohabiting Intentions | Fertility Desire | Fertility Intentions |
| --- | --- | --- | --- | --- |
|  | Coeff. (SE) | Coeff. (SE) | Coeff. (SE) | Coeff. (SE) |
| Meeting context (ref.: offline) |  |  |  |  |
| Dating app | −0.119 | 1.557** | 0.426 | 0.429 |
|  | (0.348) | (0.475) | (0.353) | (0.346) |
| Dating website | 0.318 | 0.400 | 0.388 | 0.680* |
|  | (0.372) | (0.370) | (0.336) | (0.318) |
| Other online | −0.337 | 0.338 | −0.197 | 0.110 |
|  | (0.544) | (0.471) | (0.483) | (0.386) |
| Pseudo R-squared | 0.180 | 0.194 | 0.383 | 0.162 |
| *N* | *1,281* | *761* | *1,298* | *1,297* |

Source: EFG 2018.

Note: Weighted data by wecritpers. The models control for: gender, (same-sex couple), (type of union), age, tertiary education, whether employed, whether previously married, prior cohabitation, biological children, (common children), religiosity, migration background, work-life conflict, relationship satisfaction, and partnership duration. Covariates within parenthesis are not included in all the analyses (e.g., same-sex couple not included in the model of marital intentions).

* *p* < 0.05; ** *p* < 0.01.


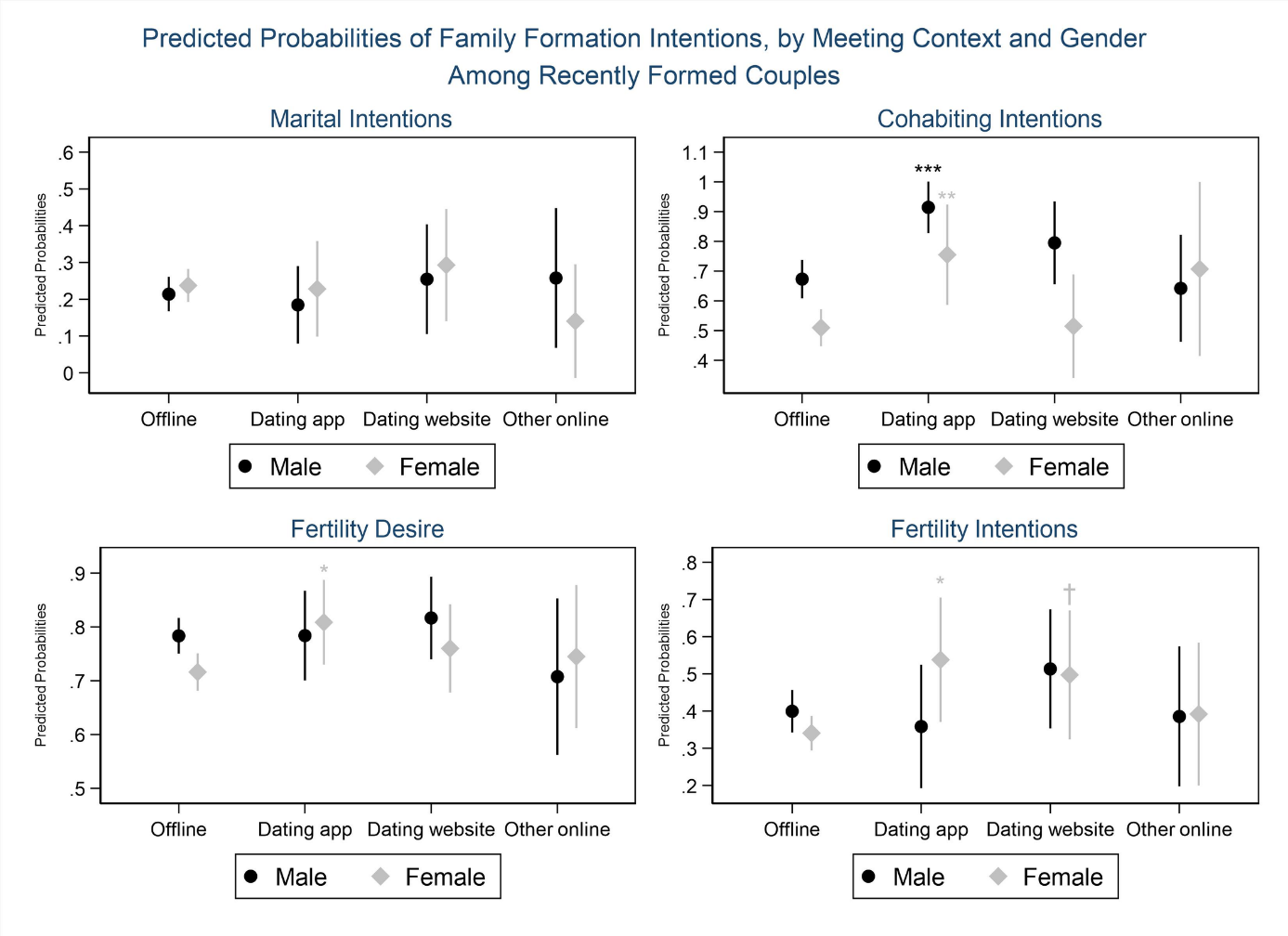


**Figure S6.2:** Predicted probabilities of family formation intentions, by meeting context and gender, 95% CI.

*Note*: Based on logistic regression models including interactions between meeting context and gender, controlling for whether same-sex couple, type of union, age, tertiary education, whether employed, whether previously married, prior cohabitation, biological children, (common children), religiosity, migration background, work-life conflict, relationship satisfaction, poor health, and partnership duration. Stars indicate probabilities significantly different compared to the offline category: † *p* < 0.10; * *p* < .05; ** *p* < 0.01; *** *p* < 0.001.

**Table S6.2: OLS regression coefficients predicting relationship and life satisfaction by meeting context -among recently formed couples**

|  | Relationship Satisfaction | Life  Satisfaction |
| --- | --- | --- |
|  | Coeff. (SE) | Coeff. (SE) |
| Meeting context (ref.: offline) |  |  |
| Dating app | −0.110 | −0.304 |
|  | (0.240) | (0.237) |
| Dating website | 0.256† | 0.191 |
|  | (0.150) | (0.166) |
| Other online | −0.419 | −1.021*** |
|  | (0.343) | (0.296) |
| R-squared | 0.100 | 0.236 |
| *N* | *1,574* | *1,578* |

Source: EFG 2018.

Note: Weighted data by wecritpers. The models control for: gender, same-sex couple, type of union, age, tertiary education, whether employed, whether previously married, prior cohabitation, biological children, religiosity, migration background, work-life conflict, poor health, and partnership duration.

† *p* < .10; *** *p* < 0.001.

**Table S6.3:** **Logistic and multinomial logistic regression coefficients predicting exogamy by meeting context - among recently formed couples**

|  | Educational Exogamy | Exogamy on Origin among Natives | Exogamy on Origin among Migrants | Age Difference  (Ref.: Age Hypergamy) | | Geographical Exogamy  (Ref.: Short Distance) | |
| --- | --- | --- | --- | --- | --- | --- | --- |
|  | Logit | Logit | Logit | Multinomial Logit | | Multinomial Logit | |
|  |  |  |  | Age Homogamy | Age Hypogamy | Moderate Distance | Long  Distance |
|  | Coeff. (SE) | Coeff. (SE) | Coeff. (SE) | Coeff. (SE) | Coeff. (SE) | Coeff. (SE) | Coeff. (SE) |
| Meeting context (ref.: offline) |  |  |  |  |  |  |  |
| Dating app | 0.601† | 0.205 | 0.568 | −0.295 | −0.291 | 0.856† | 1.077* |
|  | (0.351) | (0.394) | (0.652) | (0.357) | (0.433) | (0.462) | (0.464) |
| Dating website | 0.143 | 0.334 | 0.162 | 0.527* | 0.174 | 0.554 | 1.645*** |
|  | (0.287) | (0.339) | (0.550) | (0.258) | (0.387) | (0.460) | (0.455) |
| Other online | −0.505 | 1.489** | −0.403 | 0.364 | −0.269 | 0.111 | 0.761 |
|  | (0.427) | (0.494) | (0.522) | (0.382) | (0.644) | (0.622) | (0.594) |
| (Pseudo) R-squared | 0.0554 | 0.101 | 0.0944 | 0.0587 | | 0.0783 | |
| *N* | *1,234* | *1,036* | *529* | *1,546* | | *759* | |

Source: EFG 2018.

Note: Weighted data by wecritpers. The models control for: gender, (same-sex couple), (type of union), age, tertiary education, whether employed, previously married, prior cohabitation, biological children, religiosity, (migration background), type of residential region, work-life conflict, and partnership duration. Covariates within parenthesis are not included in all the analyses (e.g., same-sex couple not included in the model of age difference).

† *p* < .10; * *p* < 0.05; ** *p* < 0.01; *** *p* < 0.001.

## References

1. Gerlitz Y, Schupp J. Zur Erhebung der Big-Five-basiertenPersönlichkeitsmerkmale im SOEP [Assessment of Big Fivepersonality characteristics in the SOEP]. German Institute of Economic Research (Research Notes 4); 2005.
